# Decoding the similarities and differences among mycobacterial species

**DOI:** 10.1371/journal.pntd.0005883

**Published:** 2017-08-30

**Authors:** Sony Malhotra, Sundeep Chaitanya Vedithi, Tom L. Blundell

**Affiliations:** Department of Biochemistry, University of Cambridge, Cambridge, United Kingdom; Beijing Institute of Microbiology and Epidemiology, CHINA

## Abstract

Mycobacteriaceae comprises pathogenic species such as *Mycobacterium tuberculosis*, *M*. *leprae* and *M*. *abscessus*, as well as non-pathogenic species, for example, *M*. *smegmatis* and *M*. *thermoresistibile*. Genome comparison and annotation studies provide insights into genome evolutionary relatedness, identify unique and pathogenicity-related genes in each species, and explore new targets that could be used for developing new diagnostics and therapeutics. Here, we present a comparative analysis of ten-mycobacterial genomes with the objective of identifying similarities and differences between pathogenic and non-pathogenic species. We identified 1080 core orthologous clusters that were enriched in proteins involved in amino acid and purine/pyrimidine biosynthetic pathways, DNA-related processes (replication, transcription, recombination and repair), RNA-methylation and modification, and cell-wall polysaccharide biosynthetic pathways. For their pathogenicity and survival in the host cell, pathogenic species have gained specific sets of genes involved in repair and protection of their genomic DNA. *M*. *leprae* is of special interest owing to its smallest genome (1600 genes and ~1300 psuedogenes), yet poor genome annotation. More than 75% of the pseudogenes were found to have a functional ortholog in the other mycobacterial genomes and belong to protein families such as transferases, oxidoreductases and hydrolases.

## Author summary

Members of the Mycobacteriaceae family, which are known to adapt to different environmental niches, comprise bacterial species with varied genome sizes. They are unique in their cell-wall composition, which is remarkably thick and lipid-rich as compared to other bacteria. We performed a comparative analysis at the proteome level for ten mycobacterial species that differ in their pathogenicity, genome size and environmental niches. A total of 1080 orthologous clusters with representation from all ten species were obtained, and these were further examined for their domain annotations, domain architecture similarities and enriched GO terms. These core orthologous clusters are enriched in various biosynthetic pathways. The proteins that are specific to each of the ten species were also investigated for their GO functions. The *M*. *leprae* genome has a large number of pseudogenes and we searched for their functional orthologs in other mycobacterial species in order to understand the functions that are lost from the *M*. *leprae* genome. The proteins present exclusively in *M*. *leprae* genome were studied in more detail, in order to predict putative drug targets and diagnostic markers. These findings, which have implications in understanding evolution of mycobacterial genomes, identify species-specific proteins that have potential for use in developing new diagnostic tools and therapeutics.

## Introduction

*Mycobacteriacea* are known etiological agents for a variety of human infections and are broadly classified as *Mycobacterium tuberculosis* (*M*. *tuberculosis*) complex (MTBC) and Non-Tuberculous Mycobacteria (NTM). The MTBC includes several pathogenic species including *M*. *tuberculosis* that causes tuberculosis (TB) in ~10.4 million people across the globe each year. In the year 2015, ~1.4 million deaths were reported due to TB and additionally, 0.4 million deaths occurred as a result of TB infection in HIV patients [1]. Other obligate intracellular pathogenic species include *M*. *leprae* that causes leprosy in ~200,000 people annually and is mainly confined to endemic countries in the tropical zones [2]. NTMs on the other hand cause opportunistic infections and are a growing concern for a plethora of varied atypical systemic infections [3]. Currently there are more than 140 species of NTMs, some of which lead to pulmonary diseases, otitis media, osteomyelitis, lymphadenitis and skin and soft tissue infections (SSTIs) in humans [4]. One of the NTM that deserves a specific mention is a free-living rapidly growing species, *M*. *abscessus*, which is regarded as a new antibiotic nightmare that causes opportunistic infections in patients with cystic fibrosis or chronic pulmonary disease, and/or skin and soft-tissue infection [5].

The availability of the genome sequence data for several mycobacterial species, together with a variety of bioinformatics software and methods for genome analysis, makes it feasible for researchers to annotate genomes and collate information related to evolutionary traits, sequence homology, conserved regions, domain architecture, structural properties of gene products and gene ontology (GO) content. Comparative functional annotation of proteins from the genome sequencing data for pathogenic and non-pathogenic mycobacterial species can provide information related to phylogeny, frequency & distribution of orthologous protein clusters (clusters of gene families obtained from sequence comparisons of multiple species that usually reflect common functions), overlap between functional networks and species specific unique gene products [6]. This information is vital for identifying potential drug targets and unique regions/gene products that provide opportunities for developing effective diagnostic tools with considerable sensitivity and specificity.

The resulting mycobacterial genome annotations also provide an extremely useful resource for understanding strain variation and pathogenicity. The emergence of multidrug resistant and extremely drug-resistant strains underlines the need to understand orthologous genes and to identify potentially druggable targets. Earlier attempts to compare mycobacterial genomes provided information about pairwise whole-genome similarities and their predicted proteomes [7]. Since the determination of the complete genome sequence of *M*. *tuberculosis*, there have been efforts to develop inventories that record information on open reading frames (ORFs) annotations and gene expression [8–11], drug resistance mutations and drug targets [12–15], phylogenetic relationships [16,17], pathogenomics, and structure and function annotation of the mycobacterial genome [18,19].

In the current study, we have chosen ten different species—*M*. *tuberculosis*, *M*. *abscessus*, *M*. *leprae*, *M*. *marinum*, *M*. *avium*, *M*. *kansasii*, *M*. *thermoresistible*, *M*. *smegmatis*, *M*. *ulcerans* and *M*. *vanbaalenii*—for comparative analysis of genomes and protein functions. The set being investigated encompasses pathogens, opportunist pathogens and non-pathogenic species. Here we describe the mapping of orthologous clusters across the species in terms of their gene products to identify conserved regions and species-specific unique proteins from the predicted proteomes. Further, phylogenetic linkages are defined and GO annotations assessed to identify functional similarities and differences between protein targets from various species.

Of these mycobacterial species, *M*. *leprae* is under-represented in most of the known mycobacterial databases and comparative genome studies. It has the smallest genome (due to reductive evolution) among known mycobacterial genomes and a limited set of predicted proteins while half of its genome is occupied by pseudogenes [20]. Here we describe the search for functional orthologs of the pseudogenes in other mycobacterial species, to gain insight into the set of functions lost from the *M*. *leprae* genome. *M*. *leprae* has a genome size of 3,268,210 bp with only ~1600 genes, of which 22% are hypothetical proteins with unknown functions [20]. This poor annotation is also reflected by just a handful of solved protein structures (13 structures as of 23^rd^ April 2017) in the Protein DataBank (PDB). Further, it poses clinical challenges as it has a very long generation time of 14 days, is an unculturable pathogen that lacks reliable and specific molecular markers for diagnosis of the disease. Here we report a study of the unique proteins present in the genome of *M*. *leprae* for their GO functions, subcellular and transmembrane localization, gene expression profiles from a GEO dataset, essentiality, virulence and the presence of human orthologs.

## Methods

### Mycobacterial genomes

The ten mycobacterial genomes (Table 1) investigated in the present study were downloaded from UniProt [21]. These mycobacterial species cover different genome sizes and environmental niches. In the pathogenic group, we included the most virulent mycobacteria [22]: *M*. *tuberculosis*, *M*. *leprae*, *M*. *marinum* (infects broader variety of hosts and causes lesions characterized by granulomas) and *M*. *ulcerans* (causes third-most common mycobacterial disease after tuberculosis and leprosy). The opportunist pathogenic group includes NTMs that cause pulmonary and other peripheral infections in immunocompromised individuals. These definitions are adapted from an earlier comparative study of metabolic pathways of the mycobacterial species [22].

**Table 1:** Mycobacterium species used in the study and properties of their genomes.

Mycobacterium sp	Strain used	Uniprot Accession	Genome Size (bp)	No of proteins (chromosomal)	Nature of species/virulence
*M*. *tuberculosis*	h37rv	UP000001584	4,411,532	3993	Pathogen
*M*. *leprae*	TN	UP000000806	3,268,203	1603	Pathogen
*M*. *ulcerans*	agy9	UP000000765	5,631,606	4131	Pathogen
*M*. *marinum*	M	UP000001190	6,636,827	5389	Pathogen
*M*. *smegmatis*	mc2155	UP000000757	6,988,209	6601	Non-pathogenic
*M*. *vanbaalenii*	pyr1	UP000009159	6,491,865	5902	Non-pathogenic
*M*. *thermoresistibile*	ATCC19527	UP000004915	unassembled WGS	4612	Non-pathogenic
*M*. *avium*	104	UP000001574	5,475,491	5040	Opportunistic pathogen
*M*. *abscessus*	ATCC19977	UP000007137	5,067,172	4918	Opportunistic pathogen
*M*. *kansasii*	ATCC12478	UP000017786	6,432,277	5689	Opportunistic pathogen

### Identification of orthologs

Orthologs were identified in the ten species using ProteinOrthov5 [23]. Sets of orthologs that are shared across all species and between a given pair of species were identified. The remaining sets of proteins from each species that failed to identify an ortholog in any other nine species were marked as species-specific proteins. The clusters of orthologs that have representation from all the ten species are called core orthologs (gene families present in all ten species).

### Domain composition and architecture similarity in orthologs

For all the orthologous clusters identified, irrespective of the number of genes and species, we looked at the domain composition and architectural similarities in order to identify the functional similarities at the genome level.

For calculating the similarity in domain composition, for a given cluster, all pairwise orthologs were considered and were assigned Pfam domains using hmmscan from the HMMER3 package [24] and Pfam v30 database [25] at an E-value threshold of 10^−3^. For each of these pairs having representation in two species, we then calculated the fraction of shared domains, known as the domain composition similarity score (DCS, Eq 1), which can range from 0 to 1, where 1 indicates that the given pair of orthologs has exactly the same domain composition and a score close to zero reflects poor similarity in domain composition. If there are in-paralogs in the orthologous cluster, then the presence of a Pfam domain in at least one of these is sufficient to be included in the count as a shared domain.

DCS= sd12N (1)

where sd_12_ is the number of shared domains between protein p1 and p2 and N is the total number of non-redundant domains in p1 and p2.

The second level of similarity is more stringent as it considers both the order and the content of domains and is called the domain architecture similarity score (DAS, Eq 2). DAS, adapted from Forslund *et al*. [26], is calculated for each pair in the orthologous cluster and considers the number of identical aligned Pfam domains compared to the total number of domains in the pair.

DAS= al12N (2)

where al_12_ is the number of domains that are aligned with an identical domain in two given proteins p1 and p2 and N is the total number of domains in the two given proteins.

### Gene ontology (GO) term enrichment analysis

We mapped both core orthologs and species-specific sets of proteins to GO terms in all the three domains (biological process, molecular function and cellular component) by considering the following evidence codes as reliable: IMP: Inferred by Mutant Phenotype, IGI: Inferred by Genetic Interaction, IPI: Inferred by Physical Interaction, IDA: Inferred by Direct Assay, IEP: Inferred by Expression Pattern, ISS: Inferred by Structure/Sequence Similarity, TAS: Traceable Author Statement and IC: Inferred by Curation.

In order to reduce the number of GO terms and map them to broader categories, we used GOSlimViewer from AgBase [27] to map the set of proteins to GO Slim terms (broader versions of the GO ontologies that provide a summary of results of GO annotation).

To identify the GO terms enriched in a specific subset of interest, hypergeometric probabilities were calculated as:

P= CxMCn−xN−MCnN (3)

where M is the total number of GO terms in the subset, N is total number of GO terms full set, n and x is the occurrence of a GO term of interest in the full set and the subset respectively. To identify significantly enriched GO terms in the core orthologous set, p-values were calculated using hypergeometric distribution. The GO terms were considered enriched if the p-values were less than 0.05.

### Pseudogenes in Mycobacterium leprae

We used the nucleotide sequence of 1320 pseudogenes present in *M*. *leprae* and performed BLASTX against the remaining nine-mycobacterial proteomes to determine whether there is a functional ortholog present. To gain insights into the functions of these lost genes, we mapped the functional orthologs of pseudogenes identified in the *M*. *tuberculosis* genome to the protein families

### Species specific proteins

The species-specific proteins were further mapped to their GO functions to identify the enriched GO functions for all ten species using Eq 3. The *M*. *leprae* species-specific proteins were studied in detail in order to explore their potential to be used either as diagnostic markers or new drug targets. The linear B-cell epitopes for these specific proteins using BepiPred (at a threshold of 0.35) and selected the ones that are between 10–30 amino acids in length.

## Results and discussion

### Mycobacterial genomes

**Fig 1:**
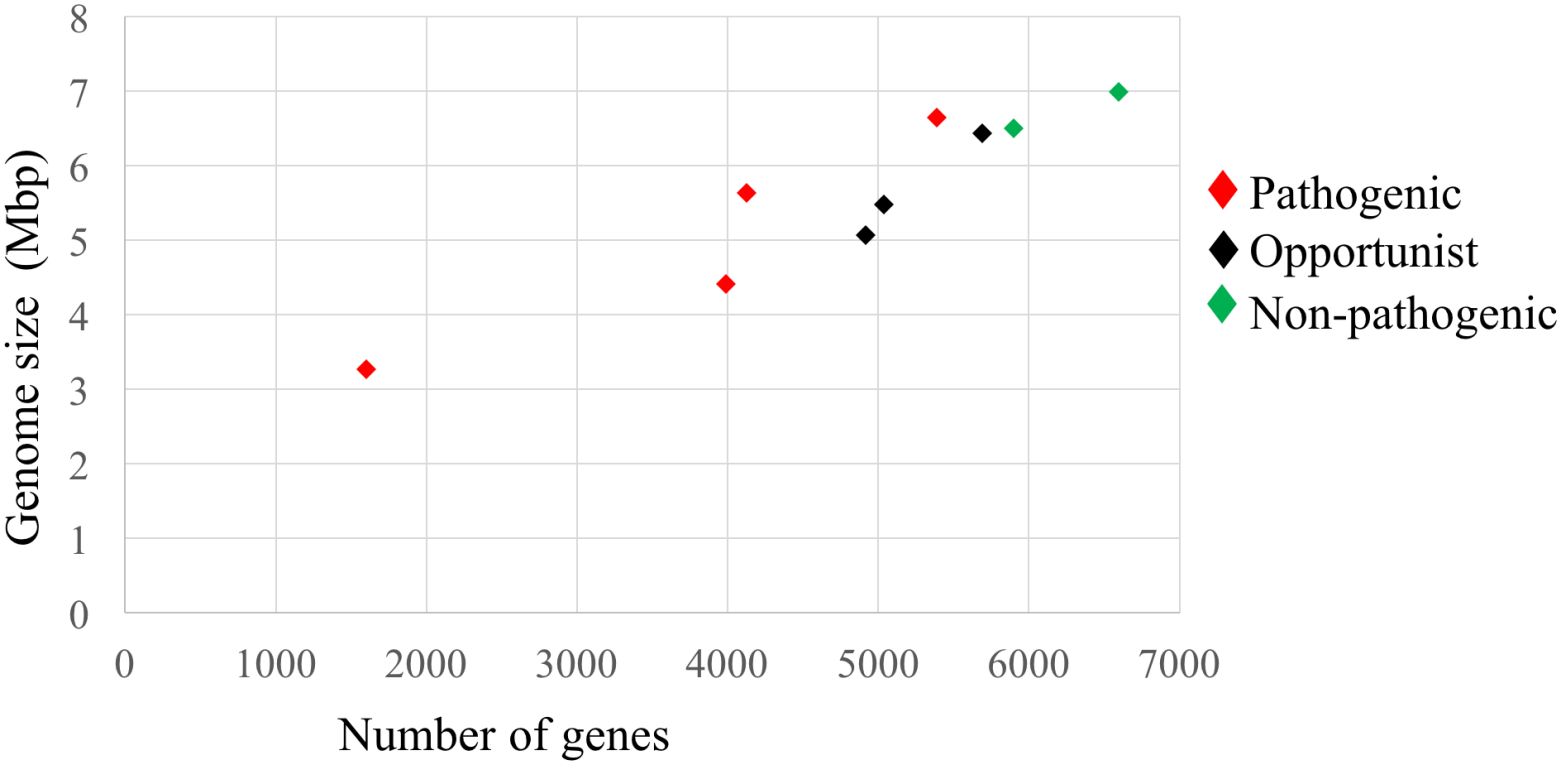
The sizes of mycobacterial genomes in relation to the number of genes. The pathogenic mycobacterial species are shown in red, in black are the species that cause opportunistic infections and non-pathogenic species are shown in green. *Mycobacterium leprae’s* genome has undergone reductive evolution and has the smallest genome, whereas the free-living *Mycobacterium smegmatis* has the largest genome.

The genome sizes of the two obligate pathogenic species, *M*. *tuberculosis and M*. *leprae*, are smaller than those of the free living non-pathogenic and opportunist pathogenic genomes (Fig 1). This is in agreement with previous observations of genome reduction and loss of genes when free-living bacteria adapt to an obligate pathogenic lifestyle [28]. However, *M*. *marinum*, a pathogen that can cause tuberculosis-like infections in aquatic organisms (fishes and amphibians) and can also cause peripheral disease characterized by granulomas in humans, has retained a higher genome size and a larger number of genes. This can be explained by its ability to infect broader range of hosts and its capacity to survive outside the host. Also, its genome is reported to have large number of polyketide synthases and non-ribosomal peptide synthases, PE and PPE proteins, secretion system proteins [29].

### Orthologous clusters

**Fig 2:**
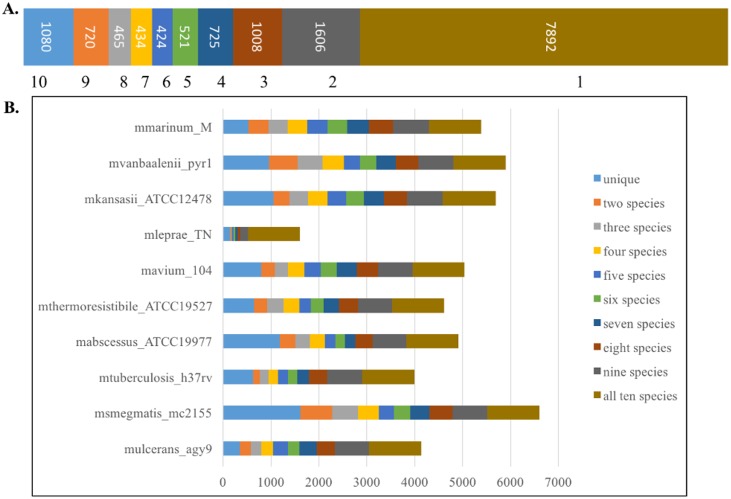
Orthologous clusters in ten species. A. The number of orthologous clusters having representation from different mycobacterial species. The core orthologs with representation from all ten species form 1080 clusters, while there are 7892 proteins from all ten species which are species-specific and do not find orthologs in any other species. B. Species-wise representation of orthologous clusters. The bar proximal to the y-axis corresponds to the number of species-specific proteins, whereas the most distal bar represents the number of proteins shared across all species.

We identified 6983 orthologous clusters that have representation from at least two of the ten species. Of these 6013 were single gene clusters (one-to-one orthologs), whereas remaining 970 clusters had in-paralogs. There were 1080 clusters that have representation of all the species and the proteins forming these clusters are labeled as core orthologs (Fig 2).

The orthologous clusters shared between any two-mycobacterial genomes were also recorded in order to identify closely related mycobacterial genomes (S1 Table). Among the pathogenic species, *M*. *tuberculosis*, *M*. *leprae and M*. *ulcerans* share the maximum number of orthologous pairs with another pathogenic species *M*. *marinum*, which has a remarkably larger genome (as discussed above). Consistent with its ability to live without the host, *M*. *marinum* shares maximum similarity with an opportunist pathogenic species *M*. *kansasii*. For the non-pathogenic species, the maximum similarity was shared within the other non-pathogenic species. The opportunist pathogenic species *M*. *abscessus* is observed to share maximum similarity with a free-living non-pathogenic species *M*. *smegmatis*. These observations correlate with the environmental niches of these species and that they have preserved the higher number of genes and have also acquired genes through horizontal gene transfer unlike pathogenic species, which have adopted an evolutionary route to minimalism (and genome reduction) to maintain their growth efficiency and competitiveness inside the host.

As *M*. *leprae* has the smallest genome with only 1600 protein coding genes, we excluded the genome of *M*. *leprae* and then repeated the ortholog identification step for the remaining nine species (S1 Fig). Although this was not observed to increase the number of orthologous clusters, the number of core orthologs (gene families present in nine mycobacterial species) increased by 40% (10,910 proteins from all ten species vs. 15,043 proteins from all nine species- excluding *M*. *leprae*).

#### Domain composition and architecture

**Fig 3:**
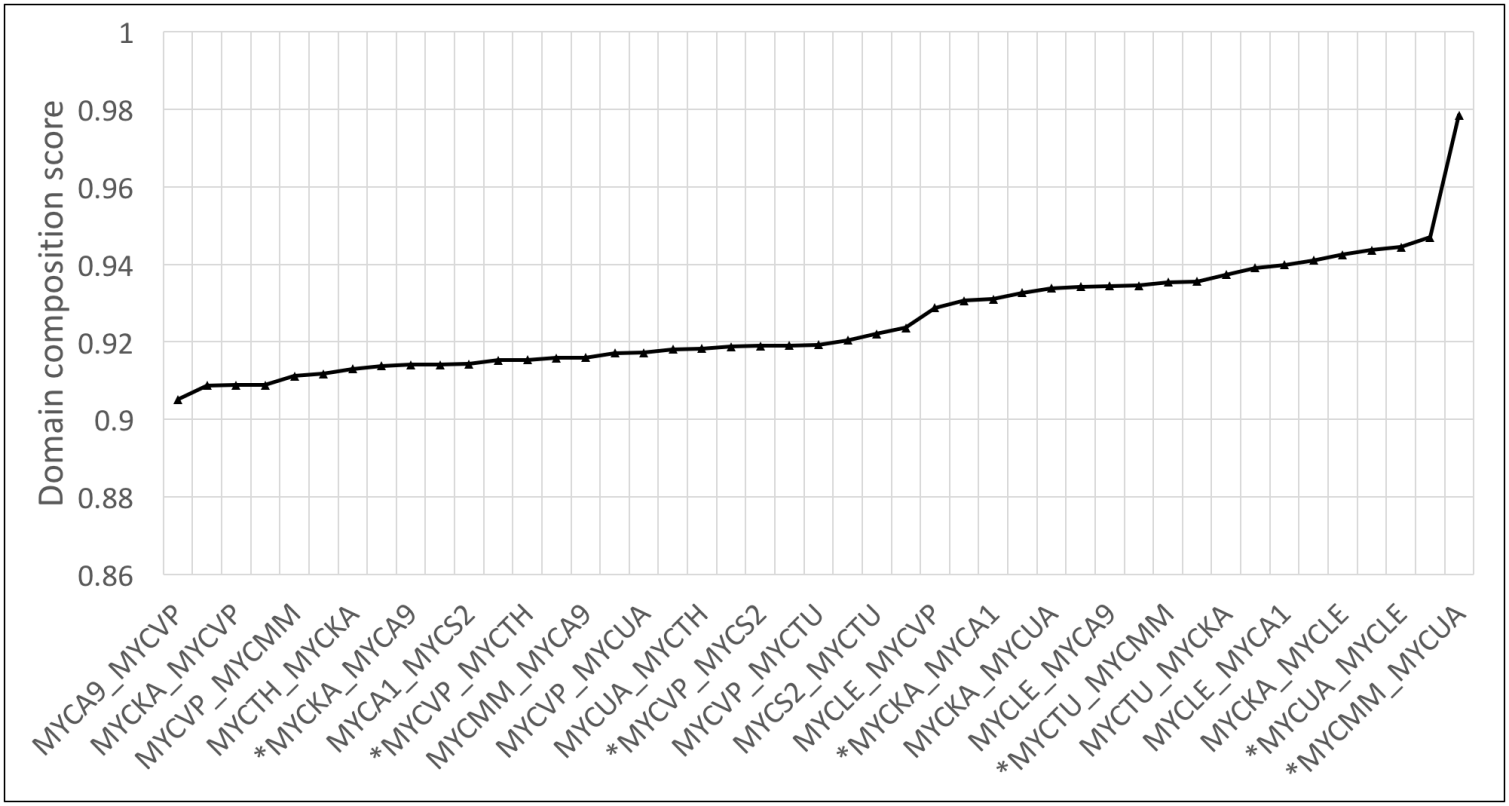
Sharing of domains between orthologs. For all the pairwise species, the average domain composition score is plotted. MYCTU: *M*.*tuberculosis*, MYCS2: *M*. *smegmatis*, MYCUA: *M*. *ulcerans*, MYCA9: *M*. *abscessus*, MYCTH: *M*. *thermoresistible*, MYCA1: *M*. *avium*, MYCLE: *M*.*leprae*, MYCKA: *M*. *kansasii*, MYCVP: *M*. *vanbaalenii*, MTCMM: *M*. *marinum*. In asterisk are the species that belong to same class namely: pathogenic and pathogenic, opportunists and opportunists, non-pathogenic and non-pathogenic.

Upon identifying orthologous clusters, we assessed the functional similarities within these clusters by investigating Pfam domain compositions for pairwise alignments (S2 Fig and Fig 3). To address the differences in the numbers of orthologous clusters, we calculated the average of the domain composition scores for each pair of species. As expected, pathogenic species have retained maximum functional similarities among themselves as they have adapted themselves to a host-dependent lifestyle. Conversely, opportunists and non-pathogenic species were less similar in terms of domain composition of orthologs reflecting less evolutionary selection of common domains and genes. The orthologs of *M*. *marinum* and *M*. *ulcerans* were more closely related with a maximum domain composition overlap (fraction of shared domains) of 0.98, which supports their evolution from a recent common ancestor [30].

**Fig 4:**
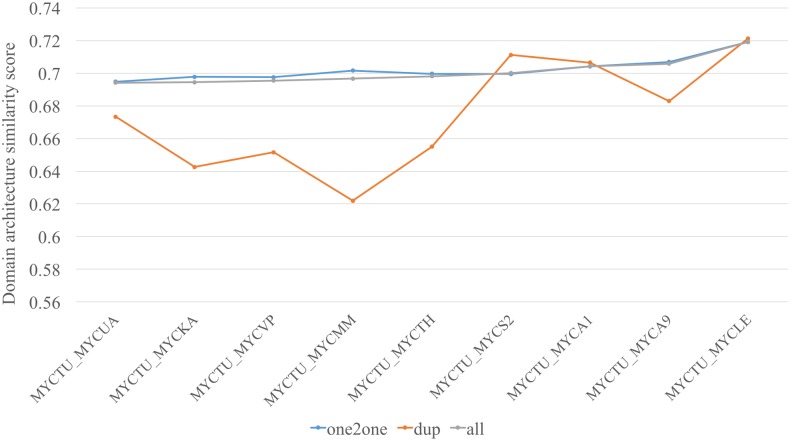
Conserved domain order in orthologs. The average domain architecture similarity score for *M*. *tuberculosis* with all other nine species. The score is higher where there is no gene duplication as compared to when in-paralogs are present. MYCTU: *M*.*tuberculosis*, MYCS2: *M*. *smegmatis*, MYCUA: *M*. *ulcerans*, MYCA9: *M*. *abscessus*, MYCTH: *M*. *thermoresistible*, MYCA1: *M*. *avium*, MYCLE: *M*.*leprae*, MYCKA: *M*. *kansasii*, MYCVP: *M*. *vanbaalenii*, MTCMM: *M*. *marinum*.

We also looked at the conservation of domain order in orthologous pairs. Fig 3 highlights this for *M*. *tuberculosis*, where the average domain-architecture similarity scores are plotted for comparison with nine other species. We calculated DAS for all orthologous clusters, one-to-one clusters (no in-paralogs (duplicated in the same genome)) and duplicated clusters (in-paralogs present). The one-to-one clusters (orthologs) retain more conserved domain architecture as compared to the in-paralogs as seen in Fig 4, which reflects that orthologs are under stronger evolutionary selection pressure than the paralogs to retain the same functions [26]. The orthologs from two pathogenic species *M*. *leprae* and *M*. *tuberculosis* were observed to have the highest domain architecture similarity indicating the functional similarities, as described later.

#### Functional annotation of core orthologs and species-specific proteins

We selected the proteins from ten species that form the 1080 orthologous clusters (10,980 proteins) and studied these for the presence of enriched GO terms using the total number of proteins in all ten species as a background. Table 2 lists the GO Slim terms. Full lists of GO functions, which are clubbed into these broader GO Slim terms, are provided as S2 Table.

**Table 2:** Function annotation of orthologs common to all species. The core orthologs from all ten mycobacterial species were examined to identify the enriched GO terms.

GO Slim ID	GO Slim Term	pvalue
**Biological process**		
GO:0006520	cellular amino acid metabolic process	2.31E^-125^
GO:0009058	biosynthetic process	1.25E^-100^
GO:0009987	cellular process	5.06E^-70^
**Cellular compartment**		
GO:0005622	intracellular	3.39E^-86^
GO:0005623	cell	1.71E^-18^
GO:0005694	chromosome	2.73E^-12^
**Molecular function**		
GO:0005198	structural molecule activity	1.72E^-197^
GO:0005488	binding	2.01E^-26^
GO:0005515	protein binding	7.81E^-14^
GO:0008565	protein transporter activity	8.24E^-32^
GO:0008907	integrase activity	3.19E^-07^
GO:0016301	kinase activity	9.71E^-09^
GO:0016740	transferase activity	8.25E^-33^
GO:0016787	hydrolase activity	6.54E^-05^
GO:0016829	lyase activity	1.36E^-13^
GO:0016853	isomerase activity	8.74E^-25^
GO:0016874	ligase activity	2.02E^-45^
GO:0030234	enzyme regulator activity	0.03

The core orthologs are enriched in functions involved in processes that are essential for the organism to grow and survive such as amino acid and purine/pyrimidine biosynthetic pathways, DNA related processes (replication, transcription, recombination and repair), RNA-methylation and modification and cell wall polysaccharide biosynthetic pathways. Many of these biosynthetic pathways such as the chorismate pathway, which is involved in biosynthesis of aromatic amino acids, are essential in mycobacterial species [31,32], which explains their enrichment in the core orthologous sets.

Apart from essentiality, another interesting observation is the existence of persistors in mycobacterial infections, as in other bacterial infections [33]. Persistors give rise to drug treatment failure and relapse of the disease apart from the growing concern of antibiotic resistant bacterial strains. The persistors are genetically similar to the drug-sensitive population but they enter the slow-growing or dormant state to evade the effects of bactericides. Recently, it has been shown that *M*. *tuberculosis* high-persistor mutants have altered biochemical pathways of amino acid and lipid biosynthesis [33–35]. This supports our observation that the proteins involved in carbon metabolism pathways are conserved across mycobacterial species and that the genes involved in biosynthetic pathways are enriched in the core orthologs. Apart from the fact that they are required for growth and survival, these genes are associated with virulence and drug evasion and hence in providing better adaptability to their respective environmental niches.

We further examined the overlap between the known and proposed drug targets for *M*. *tuberculosis* in the set of core orthologs to note the representation of these drug targets in other mycobacterial genomes. We collated the list of known potential drug targets for *M*. *tuberculosis* from several studies [36–42] including the predicted targets from the Tropical Disease Research (TDR) Targets Database [37]. ~60% of the non-redundant drug targets fall into the set of core orthologs (S3 Fig) and ~95% are present in at least one other mycobacterial genome. This set will serve as a useful and potential starting point to explore new drug targets for treating other mycobacterial infections based on their essentiality and degree of conservation in the different pathogenic mycobacterial species.

### Pathogenic *vs*. non-pathogenic genomes

In order to understand and explore the molecular and structural biology of the drug targets for pathogenic mycobacterial species such as *M*. *tuberculosis and M*. *abscessus*, non-pathogenic species (*M*. *smegmatis* and *M*.*thermoresistibile*) are usually used as surrogate systems and models in the lab [43,44]. This enables researchers to work with non-infectious strains on the bench and also *M*. *thermoresistibile* proteins can tolerate higher temperatures than the *M*. *tuberculosis* proteins and on average are more soluble [44].

To investigate the suitability of using non-pathogenic species as surrogate systems for pathogenic species, we checked the similarity of the orthologous pairs between non-pathogenic and pathogenic species (S4 Fig). The orthologs of pathogenic species (*M*. *ulcerans*, *M*. *tuberculosis*, *M*. *leprae* and *M*. *marinum*, S4A and S4B Fig in red) present in *M*. *smegmatis* and *M*. *thermoresistibile* genomes were observed to share more than 70% average percent identity. But for the opportunist pathogens (*M*. *abscessus*, *M*. *avium* and *M*. *kansasii*, S4A and S4B Fig in black), the distribution of percent identity is much wider and median is below 70% indicating that non-pathogenic species *M*. *smegmatis* and *M*. *thermoresistibile* are more suitable surrogate models for studying the proteins of pathogenic species such as *M*. *tuberculosis*.

### Pseudogenes in *M*. *leprae*

*M*. *leprae* has adapted to become an obligate pathogen and its genome has undergone a huge reduction to only 1600 protein-coding genes and large number of pseudogenes (1320) [20,45]. We inspected the other nine-mycobacterial genomes for the presence of functional orthologs of these pseudogenes in order to gain insight into what functions have been lost from the *M*. *leprae* genome during the process of genome reduction.

**Fig 5:**
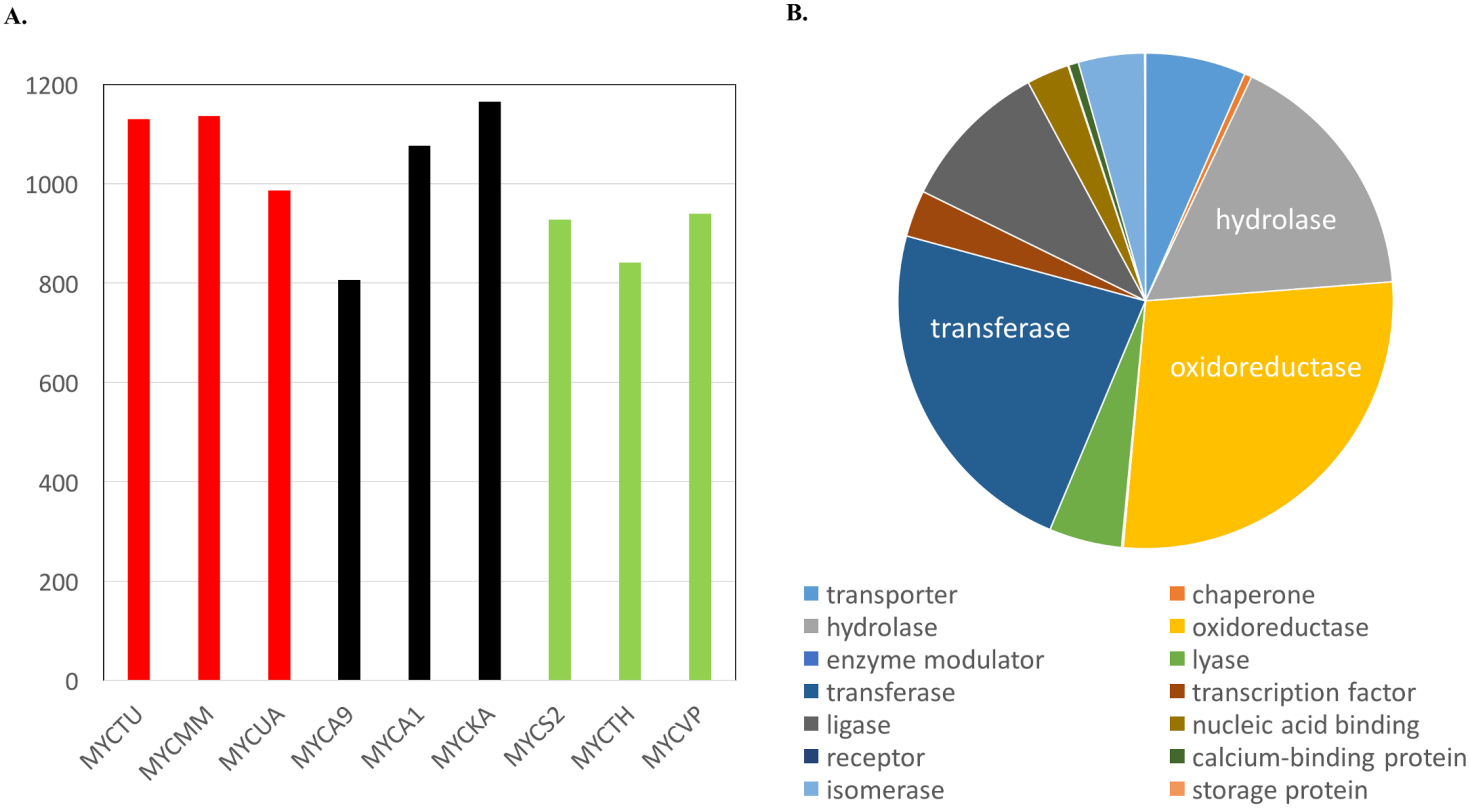
Pseudogenes in *Mycobacterium leprae* genome and their functional ortholog in other mycobacteria. A. The number of pseudogenes with a functional ortholog in other mycobacterial species. MYCTU: *M*. *tuberculosis*, MYCS2: *M*. *smegmatis*, MYCUA: *M*. *ulcerans*, MYCA9: *M*. *abscessus*, MYCTH: *M*. *thermoresistible*, MYCA1: *M*. *avium*, MYCKA: *M*. *kansasii*, MYCVP: *M*. *vanbaalenii*, MYCMM: *M*. *marinum*. B. The functional orthologs of the pseudogenes present in M. tuberculosis, mapped to the protein families to reflect their possible functions.

More than 75% of the pseudogenes were found to have a functional ortholog in the genomes of pathogenic and opportunist pathogenic species (except for *M*. *abscessus*, which had an ortholog for 61% of the pseudogenes, Fig 5A). However, the fraction of pseudogenes having a functional ortholog in non-pathogenic species was around 70% (*M*. *smegmatis*- 70%, *M*. *thermoresistible-* 64% and *M*. *vanbaalenii*- 71%).

Upon mapping the functional orthologs of pseudogenes identified in the *M*. *tuberculosis* genome to the protein families (Fig 5B), we noted that these were mainly associated with the catabolic functions such as transferases (including acetyltransferase, acyltransferase, methytransferase, transaldolase, transketolase, transaminase), oxidoreductases (including dehydrogenase and peroxidase) and hydrolases (including lipase, amylase, protease, phosphatase) thereby limiting the availability of usable energy source for *M*. *leprae* to grow. This is consistent with other studies, where they have analyzed the genome reduction and loss of functions in *M*. *leprae* genome [20,46,47]. As mentioned earlier, the proteins for all major biosynthetic pathways are fairly conserved between all mycobacterial species (as they are found in core orthologous clusters) but the energy metabolism genes appear to be more tuned to different species needs as they have evolved to survive in specific environmental niches with different growth rates.

### Species-specific proteins

We also looked at the enriched GO functions in species-specific proteins for all ten species (S3–S5 Tables). In the pathogenic species, we noticed that the genomes of *M*. *tuberculosis* and *M*. *marinum* were enriched in functions that are involved in DNA metabolism such as DNA recombination, DNA repair, DNA integration and protection (S3 Table). This supports the observation that DNA repair mechanisms are active throughout the course of tuberculosis infection as observed in infection models and clinical samples [48]. Once these pathogens infect the host, they need to survive the hostile environments inside the host cells and hence DNA repair and recombination are required to preserve the integrity of their genomes. Apart from surviving in the host-cell environment during the various stages of the infection cycle, there is a need for DNA repair and recombination mechanisms to preserve the genome during dormant phases of infection [49,50].

In the set of opportunistic pathogens (*M*. *abscessus*, *M*. *avium* and *M*. *kansasii*) specific genes, functions and processes associated with membrane transport such as the ATP-binding cassette transporter complex, high-affinity iron permease complex and oxidoreductase activity are enriched (S4 Table). The fact that the genome of *M abscessus* is known to code for many drug-efflux proteins such as ATP-binding cassette transporters and MmPL proteins [51,52] is consistent with its observed multidrug resistance. Furthermore, as these are free-living bacteria, the presence of enriched and active transport systems helps their survival through uptake of nutrients and acquisition of genes via horizontal gene transfer.

Diagnosing and treating *M*. *leprae* infections remain huge challenges due to its slow growth rate, lack of specific and reliable clinical markers and emerging drug resistance. We have therefore studied this genome in detail to identify the gene(s) that are specific to its genome, in order to identify and propose genes that can be further tested and validated for use as diagnostic markers and/or drug targets [53].

While comparing the genes of the ten-mycobacterial species, 141 *M*. *leprae* proteins were identified that lack a homolog in any of the other nine species. We further screened these 141 proteins for homologs against other mycobacterial genomes (from NCBI), and identified 86 *M*. *leprae* proteins that lack an ortholog in any other mycobacterial genomes (S5 Fig). Firstly, we scanned these 86 *M*. *leprae* specific proteins for their GO functions, transmembrane regions, presence of a human ortholog, and for their predicted essentiality, using Flux-balance analysis, from PATRIC database [10,11]) and virulence (S6 Table). Interestingly, none of the 86 had a human ortholog nor was predicted to be essential or involved in virulence. As these essentiality predictions are based on only flux-balance analysis, it would be interesting to design experiments for testing their essentiality for *M*. *leprae*.

We studied these *M*. *leprae*-specific proteins for their potential to be used either as diagnostic markers or new drug targets. Therefore, synthetic peptides presenting these epitopes could be used to raise antibodies, which can be used to detect the specific protein in a diagnostic test. Predicting the linear antigenic determinants is usually an initial step to determine antigenicity followed by prediction of non-continuous or conformational epitopes that are generally linear epitopes that are in close structural proximity upon folding. We mapped the linear B-cell epitopes for these specific proteins using BepiPred [54] (S7 Table). This is an initial step towards a search for B-cell antigens in the genome of *M*. *leprae*, which would aid to identify the antigenic determinants in this pathogenic mycobacterial species. The linear B-cell epitopes are predicted using the sequence information only; hence these provide a feasible way to run predictions at the genome-wide level, however, their accuracy rates are only about ~60–70% and more experimental validations are required to test these predictions. We could find 127 antigenic determinants in 69 of these specific proteins, which might serve as a good starting point for further experimental validations and developing diagnosis tools for leprosy (S7 Table).

In the species-specific *M*. *leprae* proteins, we investigated in detail ML2177c, which encodes for a probable uridine nucleoside phosphorylase (an important enzyme in the salvage pathway for nucleotide synthesis). This enzyme is of interest due to the following observations: a. availability of a suitable structural homolog to model the structure; b. lack of a uridine phosphorylase in *M*. *tuberculosis* genome [55–57], hence it can be specific for leprosy infection; c. known immunogenicity in both animal models and infected humans [58], which might aid in diagnosis of leprosy infection; and d. it is already being explored as a drug target for other bacterial infections such as *Salmonella typhimurium* [59]. We have also performed a transcriptomics analysis, to check the expression levels of ML2177c in patients (n = 3) with *M*. *leprae* infection from endemic regions. We measured the fold change in the expression levels of ML2177c as compared to the basal level of expression (*16S rRNA*). For two of the samples, the change of expression was two-fold and for one of the samples four-fold, indicating ML2177c is significantly expressed during leprosy infection. We also observed that ML2177c is conserved in strains of *M*. *leprae* other than TN (namely Br4923, NHDP63 and Thai).

**Fig 6:**
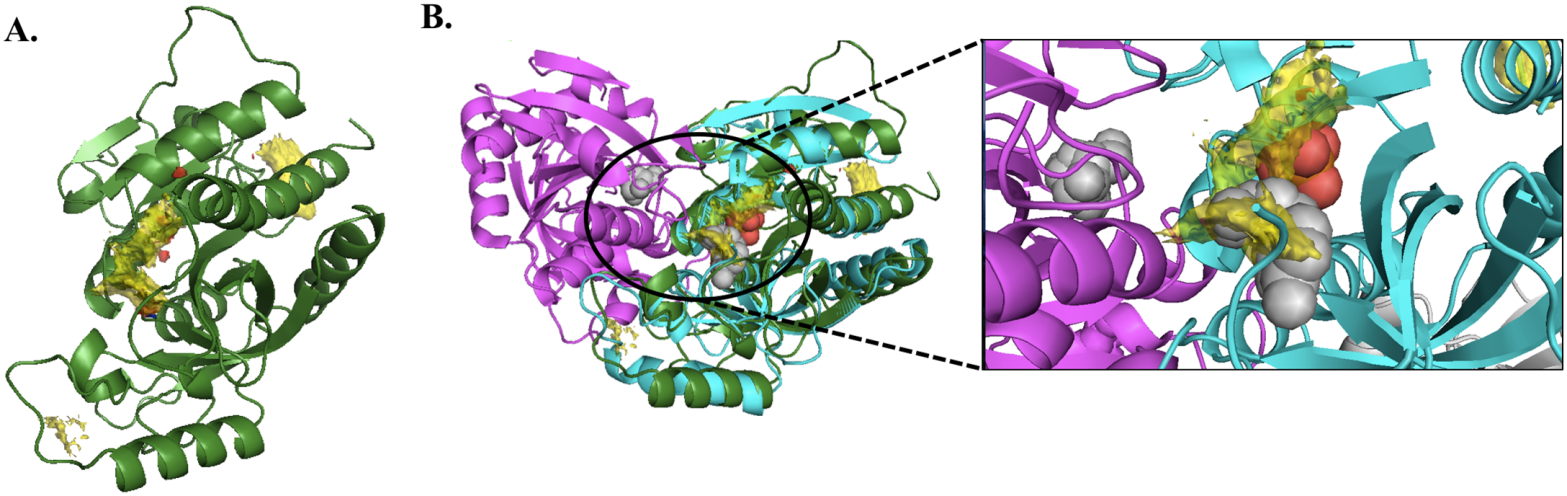
Structure modeling of *Mycobacterium leprae* specific protein (ML2177c) and predicting the druggable sites. A. Protomer from the hexameric structural model of ML2177c, with the predicted hotspots shown on the surface, in yellow where the hydrophobic fragments map, blue for hydrogen-bond donors, and red for hydrogen-bond acceptor. B. The dimer of the template (PDBID: 4R2X) is shown in cyan and magenta, and the homology model of ML2177c is in green. The inhibitor (2,2’-anhydrouridine), modeled into the dimer using the inhibitor-bound structure of uridine phosphorylase (PDBID: 3FWP), is shown in gray spheres and the phosphate is shown in red spheres.

We inspected ML2177c for its druggability by predicting the hotspots in the protein structure model. We first modeled the protein structures using our *in-house* automated modeling pipeline (*Vivace*) [18], followed by prediction of the druggable sites using our software for fragment hotspot mapping (Fig 6A) [60], which provides insights into the ligand binding site for the target. Using the known oligomeric structure for uridine phosphorylase for *Shewanella oneidensis* (PDB ID: 4R2X, 30% identity with ML2177c) as a template, we modeled the hexameric complex for ML2177c. The inhibitor 2,2'-anhydrouridine was modeled into the hexameric structure using the *S*. *typhimurium* structure (PDBID: 3FWP). The fragment hotspot maps to the dimeric interface of the modeled structure and superposes with the inhibitor-binding site, hence suggesting the druggability of ML2177c (Fig 6B).

### Conclusions

We believe that the comparative genomic studies provide insights into understanding the common mechanisms of mycobacterial pathogenesis, including pathways and functions conserved across different species. Also examining these different mycobacterial genomes for specific proteins should help distinguish the mycobacterial infection, as well as aid in designing new therapeutics and in testing some for their use in developing diagnostic kits for specific mycobacterial infections.

Here, we have highlighted functions both common and specific to different mycobacterial species. Interestingly, the drug targets predicted for *M*. *tuberculosis* were found to have orthologs in other mycobacterial genomes, suggesting their suitability as a drug target for treating other mycobacterial infections.

In our opinion, it is of value to explore the large number of pseudogenes that are retained in the M. leprae genome in more detail. Their orthologs in *M*. *tuberculosis* are reported to be non-essential but a significant proportion of these, ~43%, are observed to be expressed at different levels during different stages of disease progression [61,62]. However, the expressed pseudogenes are observed to have altered ORFs because of the large number of stop codons, the lack of start codons and their presence usually towards the end of the 3’ end of the operon. As essential and functional genes tend to be present towards the 5’ end, this appears to be an example of position-dependence of functionally significant genes [63]. The sequence comparisons of these pseudogenes in different strains of *M*. *leprae* reveal that some of these pseudogenes are strain specific, possibly implicating their role in generating genetic diversity, but more likely a reflection of selectively neutral evolution. Regarding their functions, it is proposed that they play important roles in regulation of gene expression at both transcriptional and post-transcriptional level, serve back-up functions and can be activated by phenomena such as gene conversion, regulate replication rates and rate of infection [20]. More detailed analysis on the pseudogenes in mycobacterial genomes will shed light into evolution and understanding their role during stages of infection.

## Supporting information

S1 Table Pairwise similarities between mycobacterial genomes. The matrix reflects the number of orthologous clusters having representation from any two given mycobacterial species. The maximum similarity in terms of having the maximum number of orthologous pairs between two genomes is marked in bold red and is represented row-wise.
(PDF)

S2 Table GO terms included in the GO Slim terms. For the core orthologs, we calculated the pvalues using hypergeometric distribution to find the enriched GO Slim terms. This table lists all the GO terms that are classified under the respective GO Slim terms to give a more detailed name of the GO process and function.
(XLSX)

S3 Table GO enriched terms in species-specific set of pathogenic species (*M*. *tuberculosis*, *M*. *ulcerans*, *M*. *leprae* and *M*. *marinum)*. (PDF)

S4 Table GO enriched terms in species-specific set of opportunistic species *(M*. *abscessus*, *M*. *avium* and *M*. *kansasii*). (PDF)

S5 Table Go enriched terms in species-specific set of non-pathogenic species (*M*. *smegmatis*, *M*. *thermoresistible* and *M*. *vanbaalenii*). (PDF)

S6 Table *Mycobacterium leprae* specific proteins. The unique proteins present in the genome of *M*. *leprae* mapped for their GO functions, subcellular and transmembrane localization, gene expression profiles from a GEO dataset (under-expressed in green, over-expressed in red), essentiality (flux balance based predictions), virulence and presence of human orthologs.
(PDF)

S7 Table Predicted linear B-cell epitopes in *Mycobacterium leprae* specific proteins. (PDF)

S1 Fig Orthologous clusters upon removal of *Mycobacterium leprae*. A. Species-wise representation of ortholog clusters. The bar proximal to the y-axis represents the proteins shared across all species, whereas the most distal bar represents the species-specific proteins. B. The number of genes present in the cluster that share a given number of species including and excluding *M*. *leprae*.
(TIF)

S2 Fig Domain sharing in orthologs. Domain composition scores for: A. Pathogenic vs. pathogenic species, all the scores mostly lie between 0.7–1.0, B. Opportunists vs. opportunists, C. Non-pathogenic vs. non-pathogenic. For B and C score below 0.5 are also seen. D. Pathogenic and opportunists- share high functional similarities, E. Pathogenic and non-pathogenic and F. Opportunists and non-pathogenic. MYCTU: *M*. *tuberculosis*, MYCS2: *M*. *smegmatis*, MYCUA: *M*. *ulcerans*, MYCA9: *M*. *abscessus*, MYCTH: *M*. *thermoresistible*, MYCA1: *M*. *avium*, MYCLE: *M*.*leprae*, MYCKA: *M*. *kansasii*, MYCVP: *M*. *vanbaalenii*, MTCMM: *M*. *marinum*.
(TIF)

S3 Fig Drug targets in *Mycobacterium tuberculosis* and their representation in other mycobacterial species. The representation of predicted targets for *M*. *tuberculosis* in other mycobacterial genomes.
(TIF)

S4 Fig Distribution of percent identities of orthologs of *M*. *smegmatis* and *M*. *thermoresistibile* with other pathogenic mycobacterial species. A. Box-plot showing the distribution of percent identities between the orthologs of pathogenic species (shown as red boxes) and orthologs of opportunist pathogenic species (shown as black boxes) with their orthologs in *M*. *smegmatis* genome. B. Box-plot showing the distribution of percent identities between the orthologs of pathogenic species (shown as red boxes) and orthologs of opportunist pathogenic species (shown as black boxes) with their orthologs in *M*. *thermoresistibile* genome. The abbreviations in the figure used are as follows: ulcerans_smeg: *M*. *ulcerans vs*. *M*. *smegmatis*, tb_smeg: *M*. *tuberculosis vs*. *M*. *smegmatis*, lep_smeg: *M*. *leprae vs*. *M*. *smegmatis*, mar_smeg: *M*. *marinum vs*. *M*. *smegmatis*, ab_smeg: *M*. *abscessus vs*. *M*. *smegmatis*, avium_smeg: *M*. *avium vs*. *M*. *smegmatis*, kan_smeg: *M*. *kansasii vs*. *M*. *smegmatis*. Similarly, the pairs with *M*. *thermoresistibile* (thermo).
(TIF)

S5 Fig *Mycobacterium leprae* specific proteins. The proteins identified as specific in *M*. *leprae* genome (141 proteins) were searched against other mycobacterial species’ genomes. The alignment results are plotted as sequence identity vs. query coverage. The proteins which have a hit of at least 40% query coverage and 40% sequence identity were excluded from the *M*. *leprae* species specific set (marked in red rectangle, represents 55 proteins out of 141). The remaining proteins (86) were considered for further analysis.
(TIF)
