# Supplementary material for: Decoding the similarities and differences among mycobacterial species
Source: PLoS Negl Trop Dis. 2017 Aug 30;11(8):e0005883. doi: 10.1371/journal.pntd.0005883 (PMC5595346; doi:10.1371/journal.pntd.0005883)
Supplement: S1 Table — The matrix reflects the number of orthologous clusters having representation from any two given mycobacterial species. The maximum similarity in terms of having the maximum number of orthologous pairs between two genomes is marked in bold red and is represented row-wise. (PDF) [file pntd.0005883.s001.pdf]

S1 Table. Pairwise similarities between mycobacterial genomes. The matrix reflects the number of orthologous clusters having representation from any two given mycobacterial species. The maximum similarity in terms of having the maximum number of orthologous pairs between two genomes is marked in bold red and is represented row-wise.

	MYCTU	MYCS2	MYCUA	MYCA9	MYCTU	MYCA9	MYCLE	MYCKA	MYCVP	MYCMM
**MYCTU**	-	2507	2614	2245	2341	2590	1399	2991	2533	**2999**
**MYCS2**	2507	-	2673	3028	3312	3104	1318	3111	**3995**	3181
**MYCUA**	2614	2673	-	2340	2441	2759	1356	3094	2701	**3668**
**MYCA9**	2245	**3028**	2340	-	2488	2578	1262	2584	2864	2692
**MYCTH**	2341	**3312**	2441	2488	-	2879	1285	2822	3317	2839
**MYCA1**	2590	3104	2759	2578	2879	-	1341	**3294**	3089	3261
**MYCLE**	1399	1318	1356	1262	1285	1341	-	1398	1324	**1413**
**MYCKA**	2991	3111	3094	2584	2822	3294	1398	-	3166	**3808**
**MYCVP**	2533	**3995**	2701	2864	3317	3089	1324	3166	-	3213
**MYCMM**	2999	3181	3668	2692	2839	3261	1413	**3808**	3213	-

MYCTU: *M.tuberculosis*, MYCS2: *M. smegmatis*, MYCUA*: M. ulcerans*, MYCA9: *M. abscessus*, MYCTH: *M. thermoresistible*, MYCA1: *M. avium*, MYCLE: *M.leprae*, MYCKA: *M. kansasii*, MYCVP: *M. vanbaalenii*, MTCMM: *M. marinum.*
