# Supplementary material for: Decoding the similarities and differences among mycobacterial species
Source: PLoS Negl Trop Dis. 2017 Aug 30;11(8):e0005883. doi: 10.1371/journal.pntd.0005883 (PMC5595346; doi:10.1371/journal.pntd.0005883)
Supplement: S3 Table — (PDF) [file pntd.0005883.s003.pdf]

S3 Table. GO enriched terms in species-specific set of pathogenic species (*M.tuberculosis*, *M. ulcerans*, *M.leprae* and *M. marinum).*

GO Slim term	Name	pvalue	GO terms included
M. tuberculosis			
GO:0043170	macromolecule	2.35E-05	DNA_integration
			DNA_metabolic_process
			DNA_protection
			DNA_recombinase_assembly
			DNA_recombination
			DNA_repair
			DNA_restriction-modification_system
			Mo-molybdopterin_cofactor_biosynthetic_process
			RNA_catabolic_process
			RNA_phosphodiester_bond_hydrolysis
			RNA_phosphodiester_bond_hydrolysis,_endonucleolytic
			beta-glucan_catabolic_process
			conversion_of_methionyl-tRNA_to_N-formyl-methionyl-tRNA
			cyclic_threonylcarbamoyladenosine_biosynthetic_process
			histone_H3_acetylation
			histone_acetylation
			intein-mediated_protein_splicing
			intron_homing
			mRNA_catabolic_process
			maintenance_of_CRISPR_repeat_elements
			mitotic_recombination
			molybdopterin_cofactor_biosynthetic_process
			negative_regulation_of_gene_expression
			negative_regulation_of_ribonuclease_activity
			negative_regulation_of_transcription,_DNA-templated
			negative_regulation_of_translation
			nucleic_acid_phosphodiester_bond_hydrolysis
			peptidyl-histidine_phosphorylation
			polysaccharide_catabolic_process
			positive_regulation_of_transcription,_DNA-templated
			positive_regulation_of_translation
			protein_adenylylation
			protein_autophosphorylation
			protein_phosphorylation
			protein_urmylation

			proteolysis
			rRNA_catabolic_process
			recombinational_repair
			regulation_of_gene_expression
			regulation_of_transcription,_DNA-templated
			regulation_of_translational_fidelity
			signal_transduction_by_protein_phosphorylation
			strand_invasion
			tRNA_wobble_position_uridine_thiolation
			transcription,_DNA-templated
			translational_initiation
			transposition,_DNA-mediated
GO:0050789	regulation	5.10E-07	intracellular_signal_transduction
			modulation_by_symbiont_of_host_innate_immune_response
			negative_regulation_by_symbiont_of_host_apoptotic_process
			negative_regulation_by_symbiont_of_host_innate_immune_response
			negative_regulation_of_gene_expression
			negative_regulation_of_growth
			negative_regulation_of_phosphorelay_signal_transduction_system
			negative_regulation_of_ribonuclease_activity
			negative_regulation_of_transcription,_DNA-templated
			negative_regulation_of_translation
			phosphorelay_signal_transduction_system
			positive_regulation_by_symbiont_of_host_immune_response
			positive_regulation_of_growth
			positive_regulation_of_growth_rate
			positive_regulation_of_transcription,_DNA-templated
			positive_regulation_of_translation
			regulation_of_energy_homeostasis
			regulation_of_fatty_acid_metabolic_process
			regulation_of_gene_expression
			regulation_of_protein_secretion
			regulation_of_response_to_stress
			regulation_of_transcription,_DNA-templated
			regulation_of_translational_fidelity
			signal_transduction
			signal_transduction_by_protein_phosphorylation

GO:0006139	nucleobase-containing	4.17E-07	DNA_integration
			DNA_metabolic_process
			DNA_protection
			DNA_recombinase_assembly
			DNA_recombination
			DNA_repair
			DNA_restriction-modification_system
			RNA_catabolic_process
			RNA_phosphodiester_bond_hydrolysis
			RNA_phosphodiester_bond_hydrolysis,_endonucleolytic
			conversion_of_methionyl-tRNA_to_N-formyl-methionyl-tRNA
			cyclic_nucleotide_biosynthetic_process
			cyclic_threonylcarbamoyladenosine_biosynthetic_process
			guanosine_tetraphosphate_metabolic_process
			intron_homing
			mRNA_catabolic_process
			maintenance_of_CRISPR_repeat_elements
			mitotic_recombination
			molybdopterin_cofactor_biosynthetic_process
			negative_regulation_of_ribonuclease_activity
			negative_regulation_of_transcription,_DNA-templated
			nucleic_acid_phosphodiester_bond_hydrolysis
			nucleoside_metabolic_process
			positive_regulation_of_transcription,_DNA-templated
			rRNA_catabolic_process
			recombinational_repair
			regulation_of_transcription,_DNA-templated
			strand_invasion
			tRNA_wobble_position_uridine_thiolation
			transcription,_DNA-templated
			transposition,_DNA-mediated
GO:0005576	extracellular	0.000776788	extracellular_region
GO:0009986	cell	1.77E-06	cell_surface

GO:0005515	protein	0.0029268	antisigma_factor_binding
			histone_binding
			host_cell_surface_receptor_binding
			identical_protein_binding
			protein_binding
			protein_homodimerization_activity
			toxin-antitoxin_pair_type_II_binding
			unfolded_protein_binding
GO:0016787	hydrolase	6.11E-05	ATPase-coupled_phosphate_ion_transmembrane_transporter_activity
			ATPase_activity
			ATPase_activity,_coupled_to_transmembrane_movement_of_substances
			DNA-dependent_ATPase_activity
			aminoacyl-tRNA_editing_activity
			carboxylic_ester_hydrolase_activity
			cellulase_activity
			dipeptidyl-peptidase_activity
			endodeoxyribonuclease_activity
			endonuclease_activity
			endoribonuclease_activity
			hydrolase_activity
			hydrolase_activity,_acting_on_carbon-nitrogen_(but_not_peptide)_bonds
			hydrolase_activity,_acting_on_ester_bonds
			hydrolase_activity,_acting_on_glycosyl_bonds
			hydrolase_activity,_hydrolyzing_N-glycosyl_compounds
			hydrolase_activity,_hydrolyzing_O-glycosyl_compounds
			lysophospholipase_activity
			nuclease_activity
			peptidase_activity
			phosphatase_activity
			potassium-transporting_ATPase_activity
			ribonuclease_activity
			serine-type_endopeptidase_activity
			serine-type_exopeptidase_activity
			triglyceride_lipase_activity

GO:0003676	nucleic	9.87E-05	DNA_binding
			core_promoter_proximal_region_sequence-specific_DNA_binding
			damaged_DNA_binding
			double-stranded_DNA_binding
			four-way_junction_DNA_binding
			nucleic_acid_binding
			sequence-specific_DNA_binding
			single-stranded_DNA_binding
			supercoiled_DNA_binding
M. ulcerans			
GO:0009987	cellular	0.000137717	DNA_recombination
			cellular_oxidant_detoxification
			siderophore_biosynthetic_process
			transposition,_DNA-mediated
GO:0043170	macromolecule	6.80E-26	DNA_recombination
			proteolysis
			transposition,_DNA-mediated
GO:0006139	nucleobase-containing	4.27E-33	DNA_recombination
			transposition,_DNA-mediated
GO:0003676	nucleic	6.23E-70	DNA_binding
			nucleic_acid_binding
GO:0003824	catalytic	8.87E-71	catalytic_activity
			recombinase_activity
			transposase_activity
