# Supplementary material for: Decoding the similarities and differences among mycobacterial species
Source: PLoS Negl Trop Dis. 2017 Aug 30;11(8):e0005883. doi: 10.1371/journal.pntd.0005883 (PMC5595346; doi:10.1371/journal.pntd.0005883)
Supplement: S4 Table — (PDF) [file pntd.0005883.s004.pdf]

S4 Table. GO enriched terms in species-specific set of opportunistic species *(M. abscessus, M. avium* and *M. kansasii*)**.**

GO Slim term	Name	pvalue	GO terms included
M. smegmatis			
GO:0050789	regulation	0.000722436	cell_redox_homeostasis
			negative_regulation_of_cellular_response_to_drug
			negative_regulation_of_phosphate_metabolic_process
			negative_regulation_of_transcription,_DNA-templated
			phosphorelay_signal_transduction_system
			regulation_of_quinolinate_biosynthetic_process
			regulation_of_transcription,_DNA-templated
			signal_transduction
			signal_transduction_by_protein_phosphorylation
GO:0006810	transport	7.59E-14	am+K15:K33ine_transportamine_transport
			amino_acid_transmembrane_transport
			amino_acid_transport
			carbohydrate_transmembrane_transport
			carbohydrate_transport
			divalent_inorganic_cation_transport
			ectoine_transport
			ferric_iron_transport
			galactose_transport
			gluconate_transmembrane_transport
			glycerol-3-phosphate_transport
			glycerophosphodiester_transport
			hydrogen_ion_transmembrane_transport
			ion_transport
			lactate_transmembrane_transport
			lactate_transport
			monosaccharide_transport
			nickel_cation_transmembrane_transport
			peptide_transport
			phosphate_ion_transmembrane_transport
			phosphate_ion_transport
			phosphoenolpyruvate-dependent_sugar_phosphotransferase_system
			polyamine_transmembrane_transport
			polyamine_transport
			proline_transmembrane_transport
			proline_transport

			protein_transport
			proton_transport
			sodium_ion_transport
			sulfate_transmembrane_transport
			sulfate_transport
			transmembrane_transport
			transport
GO:0016020	membrane	8.11E-05	ATP-binding_cassette_(ABC)_transporter_complex
			cell_outer_membrane
			integral_component_of_membrane
			integral_component_of_plasma_membrane
			membrane
			plasma_membrane
			pore_complex
			vesicle_membrane
GO:0005215	transporter	3.76E-08	ATPase-coupled_phosphate_ion_transmembrane_transporter_activity
			ATPase_activity,_coupled_to_transmembrane_movement_of_substances
			amino_acid-transporting_ATPase_activity
			amino_acid_transmembrane_transporter_activity
			ectoine_transmembrane_transporter_activity
			ferric-transporting_ATPase_activity
			gluconate_transmembrane_transporter_activity
			glycerol-3-phosphate-transporting_ATPase_activity
			hydrolase_activity,_acting_on_acid_anhydrides,_catalyzing_transmembrane_movement_of_substances
			lactate_transmembrane_transporter_activity
			monosaccharide-transporting_ATPase_activity
			nickel-transporting_ATPase_activity
			polar-amino_acid-transporting_ATPase_activity
			polyamine-transporting_ATPase_activity
			porin_activity
			proline:sodium_symporter_activity
			protein-N(PI)-phosphohistidine-sugar_phosphotransferase_activity
			secondary_active_sulfate_transmembrane_transporter_activity
			substrate-specific_transmembrane_transporter_activity

			sulfate_transmembrane_transporter_activity
			sulfur_carrier_activity
			symporter_activity
			taurine-transporting_ATPase_activity
			transmembrane_transporter_activity
			transporter_activity
GO:0003674	molecular_ function	1.64E-07	ATPase-coupled_phosphate_ion_transmembrane_transporter_activity
			ATPase_activity,_coupled_to_transmembrane_movement_of_substances
			amino_acid-transporting_ATPase_activity
			amino_acid_transmembrane_transporter_activity
			ectoine_transmembrane_transporter_activity
			ferric-transporting_ATPase_activity
			gluconate_transmembrane_transporter_activity
			glycerol-3-phosphate-transporting_ATPase_activity
			hydrolase_activity,_acting_on_acid_anhydrides,_catalyzing_transmembrane_movement_of_substances
			lactate_transmembrane_transporter_activity
			monosaccharide-transporting_ATPase_activity
			nickel-transporting_ATPase_activity
			polar-amino_acid-transporting_ATPase_activity
			polyamine-transporting_ATPase_activity
			porin_activity
			proline:sodium_symporter_activity
			protein-N(PI)-phosphohistidine-sugar_phosphotransferase_activity
			secondary_active_sulfate_transmembrane_transporter_activity
			sigma_factor_activity
			substrate-specific_transmembrane_transporter_activity
			sulfate_transmembrane_transporter_activity
			sulfur_carrier_activity
			symporter_activity
			taurine-transporting_ATPase_activity
			transcription_factor_activity,_sequence-specific_DNA_binding
			transmembrane_transporter_activity
M. thermoresistible			
GO:0016020	membrane	0.001604591	ATP-binding_cassette_(ABC)_transporter_complex
			integral_component_of_membrane

			membrane
			plasma_membrane
GO:0016491	oxidoreduc tase	8.93E-05	4-hydroxy-tetrahydrodipicolinate_reductase
			L-aspartate:fumarate_oxidoreductase_activity
			L-aspartate_oxidase_activity
			N,N-dimethylaniline_monooxygenase_activity
			acyl-CoA_dehydrogenase_activity
			dioxygenase_activity
			glucose-6-phosphate_dehydrogenase_(coenzyme_F420)_activity
			monooxygenase_activity
			nitrite_reductase_[NAD(P)H]_activity
			nitronate_monooxygenase_activity
			oxidoreductase_activity
			oxidoreductase_activity,_acting_on_CH-OH_group_of_donors
			oxidoreductase_activity,_acting_on_paired_donors,_with_incorporation_or_reduction_of_molecular_oxygen
			oxidoreductase_activity,_acting_on_the_CH-CH_group_of_donors
			oxidoreductase_activity,_acting_on_the_CH-OH_group_of_donors,_NAD_or_NADP_as_acceptor
			oxidoreductase_activity,_acting_on_the_aldehyde_or_oxo_group_of_donors,_NAD_or_NADP_as_acceptor
GO:0003824	catalytic	0.000309454	N-acetylmuramoyl-L-alanine_amidase_activity
			catalytic_activity
			transposase_activity
M. vanbaalenii			
GO:0050789	regulation	0.000789493	cell_redox_homeostasis
			intracellular_signal_transduction
			ionotropic_glutamate_receptor_signaling_pathway
			negative_regulation_of_phosphate_metabolic_process
			negative_regulation_of_transcription,_DNA-templated
			phosphorelay_signal_transduction_system
			regulation_of_transcription,_DNA-templated
			signal_transduction
			signal_transduction_by_protein_phosphorylation

GO:0005575	cellular_component	0.001191057	ATP-binding_cassette_(ABC)_transporter_complex
			extracellular_matrix
			integral_component_of_membrane
			integral_component_of_plasma_membrane
			periplasmic_space
			plasma_membrane
			primosome_complex
			ribosome
GO:0016020	membrane	3.32E-06	ATP-binding_cassette_(ABC)_transporter_complex
			integral_component_of_membrane
			integral_component_of_plasma_membrane
			membrane
			plasma_membrane
GO:0003676	nucleic	0.000544591	DNA_binding
			RNA_binding
			nucleic_acid_binding
			sequence-specific_DNA_binding
