# Supplementary material for: Decoding the similarities and differences among mycobacterial species
Source: PLoS Negl Trop Dis. 2017 Aug 30;11(8):e0005883. doi: 10.1371/journal.pntd.0005883 (PMC5595346; doi:10.1371/journal.pntd.0005883)
Supplement: S5 Table — (PDF) [file pntd.0005883.s005.pdf]

S5 Table. Go enriched terms in species-specific set of non-pathogenic species (*M. smegmatis*, *M. thermoresistible* and *M. vanbaalenii*).

GO Slim term	Name	pvalue	GO terms included
M. abscessus			
GO:0005575	cellular_ component	7.12E-06	ATP-binding_cassette_(ABC)_transporter_complex
			cytosol
			high-affinity_iron_permease_complex
			integral_component_of_membrane
			nucleoid
			phosphopyruvate_hydratase_complex
			plasma_membrane
			respiratory_chain
GO:0016020	membrane	6.99E-10	ATP-binding_cassette_(ABC)_transporter_complex
			high-affinity_iron_permease_complex
			integral_component_of_membrane
			membrane
			plasma_membrane
			respiratory_chain
GO:0016491	oxidoreductase activity	5.54E-05	2,3-dihydro-2,3-dihydroxybenzoate_dehydrogenase_activity
			3-hydroxyisobutyrate_dehydrogenase_activity
			4-hydroxyphenylpyruvate_dioxygenase_activity
			N,N-dimethylaniline_monooxygenase_activity
			acyl-CoA_dehydrogenase_activity
			acyl-[acyl-carrier-protein]_desaturase_activity
			dihydrolipoyl_dehydrogenase_activity
			dioxygenase_activity
			glutamate_dehydrogenase_(NAD+)_activity
			glutamate_synthase_activity
			heme_oxygenase_(decyclizing)_activity
			malate_dehydrogenase_(menaquinone)_activity
			malate_dehydrogenase_(quinone)_activity
			methylenetetrahydrofolate_reductase_(NAD(P)H)_activity
			monooxygenase_activity
			oxidoreductase_activity
			oxidoreductase_activity,_acting_on_CH-OH_group_of_donors
			oxidoreductase_activity,_acting_on_a_sulfur_group_of_donors,_NAD(P)_as_acceptor
			oxidoreductase_activity,_acting_on_a_sulfur_group_of_donors,_disulfide_as_acceptor
			oxidoreductase_activity,_acting_on_paired_donors,_with_incorporation_or_reduction_of_molecular_oxygen

			oxidoreductase_activity,_acting_on_single_donors_with_incorporation_of_molecular_oxygen
			oxidoreductase_activity,_acting_on_the_CH-CH_group_of_donors
			oxidoreductase_activity,_acting_on_the_CH-NH2_group_of_donors
			oxidoreductase_activity,_acting_on_the_aldehyde_or_oxo_group_of_donors,_NAD_or_NADP_as_acceptor
			peptide-methionine_(R)-S-oxide_reductase_activity
			peroxiredoxin_activity
			phenylacetyl-CoA_1,2-epoxidase_activity
			phosphogluconate_dehydrogenase_(decarboxylating)_activity
			pyridoxamine-phosphate_oxidase_activity
			sulfite_reductase_(NADPH)_activity
			superoxide_dismutase_activity
M. avium			
GO:0008152	metabolic	0.002461474	DNA_integration
			DNA_ligation_involved_in_DNA_repair
			DNA_metabolic_process
			DNA_methylation
			DNA_modification
			DNA_recombination
			DNA_repair
			DNA_replication
			DNA_replication,_synthesis_of_RNA_primer
			aromatic_compound_catabolic_process
			base-excision_repair
			carbohydrate_metabolic_process
			cell_wall_macromolecule_catabolic_process
			cellular_aromatic_compound_metabolic_process
			cyclic_nucleotide_biosynthetic_process
			dephosphorylation
			fatty_acid_beta-oxidation
			fatty_acid_metabolic_process
			glucose_metabolic_process
			glycolytic_process
			intein-mediated_protein_splicing
			lipid_metabolic_process
			lysine_biosynthetic_process_via_diaminopimelate
			mannitol_metabolic_process
			metabolic_process

			methylation
			nucleic_acid_phosphodiester_bond_hydrolysis
			nucleotide-excision_repair
			oxidation-reduction_process
			pentose-phosphate_shunt
			peptidoglycan_catabolic_process
			phosphatidylinositol_phosphorylation
			phosphorylation
			protein_phosphorylation
			proteolysis
			pyridoxal_phosphate_biosynthetic_process
			pyruvate_metabolic_process
			rRNA_methylation
			rRNA_modification
			regulation_of_transcription,_DNA-templated
			removal_of_superoxide_radicals
			signal_transduction_by_protein_phosphorylation
			superoxide_metabolic_process
			tRNA_thio-modification
			thiamine_biosynthetic_process
			thiamine_diphosphate_biosynthetic_process
			transcription,_DNA-templated
			transposition,_DNA-mediated
			tryptophan_catabolic_process_to_kynurenine
GO:0016020	membrane	0.000289508	ATP-binding_cassette_(ABC)_transporter_complex
			integral_component_of_membrane
			membrane
			plasma_membrane
GO:0016491	oxidoreductase	0.000170477	(+)-trans-carveol_dehydrogenase_activity
			3-hydroxyacyl-CoA_dehydrogenase_activity
			3-oxo-pimeloyl-[acp]_methyl_ester_reductase_activity
			3-oxoacyl-[acyl-carrier-protein]_reductase_(NADPH)_activity
			4-hydroxy-tetrahydrodipicolinate_reductase
			acyl-CoA_dehydrogenase_activity
			dioxygenase_activity
			glucose-6-phosphate_dehydrogenase_activity
			monooxygenase_activity

			morphine_6-dehydrogenase_activity
			oxidoreductase_activity
			oxidoreductase_activity,_acting_on_paired_donors,_with_incorporation_or_reduction_of_molecular_oxygen
			oxidoreductase_activity,_acting_on_paired_donors,_with_incorporation_or_reduction_of_molecular_oxygen,_NAD(P)H_as_one_donor,_and_incorporation_of_two_atoms_of_oxygen_into_one_donor
			oxidoreductase_activity,_acting_on_the_CH-CH_group_of_donors
			oxidoreductase_activity,_acting_on_the_aldehyde_or_oxo_group_of_donors,_NAD_or_NADP_as_acceptor
			peroxiredoxin_activity
			protein_disulfide_oxidoreductase_activity
			pyridoxamine-phosphate_oxidase_activity
			superoxide_dismutase_activity
M. kansasii			
GO:0005575	cellular_component	0.001156494	ATP-binding_cassette_(ABC)_transporter_complex
			integral_component_of_membrane
			nucleoid
			plasma_membrane
			respiratory_chain
GO:0016020	membrane	3.38E-05	ATP-binding_cassette_(ABC)_transporter_complex
			integral_component_of_membrane
			membrane
			plasma_membrane
			respiratory_chain
