# Supplementary material for: Decoding the similarities and differences among mycobacterial species
Source: PLoS Negl Trop Dis. 2017 Aug 30;11(8):e0005883. doi: 10.1371/journal.pntd.0005883 (PMC5595346; doi:10.1371/journal.pntd.0005883)
Supplement: S6 Table — The unique proteins present in the genome of M. leprae mapped for their GO functions, subcellular and transmembrane localization, gene expression profiles from a GEO dataset (under-expressed in green, over-expressed in red), essentiality (flux balance based predictions), virulence and presence of human orthologs. (PDF) [file pntd.0005883.s006.pdf]

**S6 Table. *Mycobacterium leprae* specific proteins.** The unique proteins present in the genome of *M. leprae* mapped for their GO functions, subcellular and transmembrane localization, gene expression profiles from a GEO dataset (under-expressed in green, over-expressed in red), essentiality (flux balance based predictions), virulence and presence of human orthologs.

| Entry | Gene names | Length | Human Homologues | Gene Expression | Gene ontology (GO) | Transmembrane |
|---|---|---|---|---|---|---|
| Q9CBI3 | ML1928 | 45 | No | -2.07 | integral component of membrane [GO:0016021] | Yes |
| Q9CCI8 | ML0777 | 64 | No | -0.49 | integral component of membrane [GO:0016021] | Yes |
| Q9CBV9 | ML1523 | 96 | No | 0.08 | integral component of membrane [GO:0016021] | Yes |
| Q9CDF0 | ML0009 | 63 | No | -0.14 | integral component of membrane [GO:0016021] | Yes |
| Q7AQ76 | ML1292 | 80 | No | 0.00 | integral component of membrane [GO:0016021] | Yes |
| Q7APW0 | ML2347 | 336 | No | 0.28 | integral component of membrane [GO:0016021] | Yes |
| Q9CCC6 | ML0958 | 143 | No | -0.68 | integral component of membrane [GO:0016021] | Yes |
| Q9CCZ1 | ML2651 | 105 | No | -0.46 | integral component of membrane [GO:0016021] | Yes |

| | | | | | | |
|---|---|---|---|---|---|---|
| Q9CBG6 | ML1990 | 80 | No | -1.27 | integral component of membrane [GO:0016021] | Yes |
| Q7AQ91 | ML1148 | 51 | No | -0.19 | | |
| Q9CC35 | ML1344 | 86 | No | -0.91 | DNA binding [GO:0003677] | |
| Q9CBR2 | ML1717 | 97 | No | -0.04 | | |
| Q9CBD1 | ML2177 | 317 | No | 0.13 | cytoplasm [GO:0005737]; uridine phosphorylase activity [GO:0004850]; nucleoside metabolic process [GO:0009116]; nucleotide catabolic process [GO:0009166] | |
| Q07297 | sra ML0411 MLCL383.14 | 408 | No | 0.18 | | |
| Q9CDF1 | ML0008 | 124 | No | -0.73 | | |
| Q9CDE2 | ML0024 | 83 | No | -0.06 | | |
| Q9CDE1 | ML0025 | 96 | No | -0.68 | | |
| Q9CDC8 | ML0070 | 87 | No | 0.11 | | |
| Q9CD77 | ML0141 | 84 | No | -0.50 | | |
| Q9CD73 | ML0152 | 62 | No | -0.85 | | |
| Q7AQN3 | ML0162 | 92 | No | -0.28 | | |
| Q9CD59 | ML0217 | 81 | No | -0.29 | | |
| Q7AQL5 | ML0218 | 128 | No | 0.02 | | |
| Q7AQK1 | ML0291 | 130 | No | -0.36 | | |
| Q9CCX2 | ML0292 | 79 | No | -1.78 | | |
| Q9CCX1 | ML0293 | 69 | No | 1.49 | | |
| Q7AQJ8 | ML0308 | 234 | No | 0.06 | | |

| | | | | | | |
|---|---|---|---|---|---|---|
| Q9CCU2 | ML0447 | 158 | No | 0.00 | heme binding [GO:0020037]; iron ion binding [GO:0005506]; oxidoreductase activity, acting on paired donors, with incorporation or reduction of molecular oxygen [GO:0016705] | |
| Q7AQJ2 | ML0448 | 90 | No | 0.34 | | |
| Q9CCT8 | ML0464 | 95 | No | -0.75 | | |
| Q7AQI7 | ML0470 | 96 | No | -0.71 | | |
| Q9CCR6 | ML0527 | 84 | No | 0.09 | | |
| Q9CCN7 | ML0568 | 197 | No | -0.91 | | |
| Q7AQI4 | ML0574 | 105 | No | -0.21 | | |
| Q7AQI3 | ML0576 | 76 | No | -0.76 | | |
| Q7AQH1 | ML0656 | 93 | No | -0.13 | | |
| Q7AQH0 | ML0659 | 108 | No | -0.19 | | |
| Q9CCM5 | ML0664 | 87 | No | -0.15 | | |
| Q9CCM3 | ML0679 | 75 | No | 0.00 | | |
| Q9CCK3 | ML0757 | 230 | No | -0.43 | | |
| Q9CCG0 | ML0837 | 127 | No | -0.68 | | |
| Q7AQE5 | ML0863 | 104 | No | -0.46 | | |
| Q9CCD5 | ML0928 | 94 | No | -0.65 | | |
| Q9CCD4 | ML0938 | 98 | No | -0.50 | | |
| Q9CCD3 | ML0939 | 80 | No | -0.13 | | |
| Q9CCD0 | ML0950 | 66 | No | -0.13 | | |
| Q9CCC7 | ML0957 | 124 | No | 0.17 | | |
| Q9CCC5 | ML0959 | 117 | No | -0.21 | | |
| Q9CCC4 | ML0963 | 154 | No | -0.04 | | |
| Q9CCC3 | ML0964 | 85 | No | -0.04 | | |
| Q9CCB7 | ML1010 | 80 | No | 0.00 | | |
| Q9CCB6 | ML1011 | 140 | No | 0.30 | | |
| Q9CCB3 | ML1018 | 71 | No | 0.15 | | |
| Q7AQA0 | ML1057 | 117 | No | -0.84 | | |
| Q9CC71 | ML1186 | 100 | No | 0.37 | | |
| Q9CC60 | ML1243 | 153 | No | 1.66 | | |
| Q9CC51 | ML1275 | 78 | No | -0.51 | | |

| | | | | | | |
|---|---|---|---|---|---|---|
| Q9CC28 | ML1384 | 112 | No | -0.59 | | |
| Q9CC03 | ML1445 | 74 | No | -0.76 | | |
| Q9CBU8 | ML1575 | 97 | No | 0.21 | | |
| Q7AQ39 | ML1601 | 121 | No | 0.15 | catalytic activity [GO:0003824]; biosynthetic process [GO:0009058] | |
| Q9CBT9 | ML1602 | 106 | No | -0.19 | | |
| Q9CBT8 | ML1603 | 86 | No | -0.77 | | |
| Q9CBT7 | ML1604 | 129 | No | -0.33 | | |
| Q9CBT6 | ML1605 | 66 | No | -0.46 | | |
| Q7AQ24 | ML1788 | 154 | No | 0.07 | | |
| Q9CBJ3 | ML1915 | 114 | No | -0.06 | | |
| Q9CBH4 | ML1976 | 115 | No | 0.34 | | |
| Q9CBH3 | ML1979 | 135 | No | 0.31 | | |
| Q9CBG7 | ML1989 | 116 | No | -0.16 | | |
| Q9CBF6 | ML2035 | 87 | No | 0.06 | | |
| Q9CBF4 | ML2044 | 73 | No | -0.67 | | |
| Q9CBD4 | ML2172 | 118 | No | 0.12 | | |
| Q9CBD2 | ML2176 | 85 | No | -1.33 | | |
| Q9CBC5 | ML2201 | 79 | No | -1.00 | | |
| Q9CBC0 | ML2244 | 113 | No | -0.27 | | |
| Q9CBB9 | ML2249 | 105 | No | -0.21 | | |
| Q7APX0 | ML2252 | 78 | No | -0.37 | | |
| Q9CBA2 | ML2283 | 104 | No | 1.04 | | |
| Q9CBA1 | ML2284 | 119 | No | -0.18 | | |
| Q49942 | ML2346 | 301 | No | 0.14 | | |
| Q9CB35 | ML2468 | 126 | No | -1.19 | | |
| Q9CD23 | ML2562 | 96 | No | -0.37 | | |
| Q9CD21 | ML2567 | 159 | No | 0.00 | | |
| Q9CCY7 | ML2666 | 84 | No | -0.56 | | |
| Q49769 | ML0638 B1937_F3_110 | 100 | No | -0.45 | extracellular region [GO:0005576] | |
