# Supplementary material for: Decoding the similarities and differences among mycobacterial species
Source: PLoS Negl Trop Dis. 2017 Aug 30;11(8):e0005883. doi: 10.1371/journal.pntd.0005883 (PMC5595346; doi:10.1371/journal.pntd.0005883)
Supplement: S7 Table — (PDF) [file pntd.0005883.s007.pdf]

S7 Table: Predicted linear B-cell epitopes in *Mycobacterium leprae* specific proteins.

M. leprae ID	Start position in sequence	End position in sequence	Length of epitope	Average score for all residues	Peptide sequence
ML0008	78	97	20	0.9276	AQTDEEVYSRPISDNTEYGV
ML0008	59	71	13	1.041461538	AGHQSSKAEGVNT
ML0024	34	45	12	0.922083333	ADAFDDKPELGN
ML0024	55	65	11	0.760818182	AAIRGAGDDCG
ML0070	20	29	10	0.6982	QHAVGGGPVV
ML0141	57	71	15	1.581	RHVPEAPRPTSHGTS
ML0141	28	37	10	0.9513	GNRGGTGMRG
ML0152	27	40	14	1.105714286	MAQANRSTDQGNNA
ML0162	34	60	27	1.068111111	DQIGNGSSSRAPSLGNPHTAVPNVMA D
ML0162	79	90	12	1.091083333	DQSGNHSDQKHV
ML0217	2	13	12	1.01675	GVGRPCAEEPQT
ML0217	22	31	10	0.9867	GLSGGAGSSA
ML0218	17	39	23	0.979086957	TLRSKDPRQGNWPVTVSSEVAAN
ML0218	94	112	19	0.819684211	KPIASSYAVGTASNSDKTI
ML0291	95	115	21	1.396380952	TPTFQPTKTTDRTKRSPRSNT
ML0291	10	28	19	1.011315789	VNAHSSSSVATDPNEVFHQ
ML0291	68	78	11	0.798545455	AHRTTETKNLS
ML0292	69	79	11	1.415181818	DPIPRAKYPGP
ML0293	7	16	10	1.041	FDDDPSKQIQ
ML0293	40	49	10	0.9472	HRQTSSGTVS
ML0308	38	58	21	0.917380952	CPGGASSATAEINAAGGRSMA
ML0308	78	94	17	0.766882353	AETDRGNAYVRAHPDEY
ML0308	101	111	11	0.790636364	SPEHHHQSRSR
ML0411	209	231	23	0.959695652	SVAQSEEHGSDSMSQSYNTCGSV
ML0411	235	255	21	1.230047619	ELCDSPFGTPSQSSQSNDLSA
ML0411	372	388	17	0.885882353	TAVSGPLEGVTQPAEEV
ML0411	185	200	16	0.70325	DSVNSFHSSSSSDSLY
ML0411	393	406	14	1.422928571	VAGGSGTGGPAFNE
ML0411	347	358	12	0.785416667	ENWATASQPVMA
ML0411	164	174	11	0.825909091	GKMENFEPAPQ
ML0411	18	27	10	1.1706	RGPGSTPLWG
ML0447	131	148	18	1.096444444	PMPGGPMLTNSTPTESCR
ML0447	4	16	13	1.269307692	SADPSTGEQLDTD
ML0447	58	68	11	0.598454545	EIYQNRSDRGI
ML0448	26	47	22	0.847818182	RPITAQQTSVSAASWYKPKGTM
ML0464	57	76	20	1.3438	DSTSVGPGPGNMPWLDDPGL

ML0527	9	21	13	0.714769231	VKGHHRQPQRALH
ML0527	44	53	10	0.9073	SIPATRTTRQ
ML0568	164	181	18	1.403388889	PGPPASVEAPPAAAPVVA
ML0568	26	43	18	0.997333333	PSAAAAPLSMPTGGPTCI
ML0568	146	156	11	1.223909091	KGVPTASPQAA
ML0576	25	35	11	1.111727273	TGNARTAEDWP
ML0638	17	42	26	0.913807692	STAGTPHAATSAAAGQYRVAATGTRP
ML0638	60	70	11	0.950909091	PRNRNHQDPWH
ML0656	71	93	23	1.192434783	VDSSQPHHQKTEISQQENNRGK
ML0659	25	46	22	1.106136364	KPGAGKLDGAKNDAGKRKKTLS
ML0659	48	58	11	0.813636364	KHSIPGRWPSA
ML0664	56	66	11	0.963363636	EGMAGKDIDPR
ML0679	60	75	16	1.023375	VTALDDRGAGLDHPSQ
ML0679	21	32	12	1.269833333	GSGSGPAAPVWP
ML0679	38	48	11	0.973727273	PAASYQPWPEQ
ML0757	189	205	17	1.232352941	PVTGRVDSNLPDPQSPL
ML0757	83	99	17	0.797705882	VRPQNFDVGYRPDGARI
ML0757	61	72	12	1.191083333	ASQLQPSQPDCV
ML0757	104	115	12	0.793	KTLNDHDSVGKN
ML0757	1	12	12	0.74575	MQPMPGINLPKD
ML0863	33	47	15	0.8016	SAPGRSEIDVEGISA
ML0928	54	67	14	1.149928571	DPENNPRLRDPQSL
ML0938	86	98	13	0.931769231	EETARNVLPEEEE
ML0939	15	28	14	0.840857143	HTWGPSKFGQSQSC
ML0950	34	51	18	1.524277778	GHAPEQRNASPSTPTGVI
ML0957	66	94	29	1.183862069	QCVDSADTPNHRGNSAGNRHPDRLRA DAA
ML0957	20	29	10	0.9448	RSGYPTRWGG
ML0959	78	100	23	0.993347826	PLPQRGESRRDPAMTMDETVASG
ML0959	39	53	15	1.213	QQEKNRPDRVDQHGN
ML0959	12	22	11	1.141545455	NIAPSNRRPAG
ML0963	1	14	14	0.891857143	MPRTGDVYGDVRRD
ML1010	25	47	23	1.835521739	AVDEDCTNAGRGPPDPPPARARV
ML1011	78	102	25	1.74124	GAVPSESAEPTCDSTAPPPANSGNH
ML1011	26	42	17	0.995823529	LGDHQTNPMTADPLTHG
ML1011	45	59	15	0.998466667	LVPLTSPSHANKGNP
ML1018	55	71	17	0.690117647	HRNEQHSVAGYASSHR
ML1057	65	88	24	1.24375	ADVAGAAKSGPVQPMGDRGSVSPV
ML1057	101	112	12	0.912333333	RAFAMPSSPAAG
ML1148	1	14	14	1.073428571	MRDEKQPFEANTAG
ML1186	1	15	15	0.841933333	MTQKDGSTLIGPAAH
ML1186	53	63	11	1.250727273	PDLPPGDRGFL

ML1243	119	129	11	1.062818182	EEKNPLAPSAK
ML1243	143	153	11	0.994636364	RTGPCRHPRRD
ML1275	60	75	16	0.665	TWSSSLTSSQTQWHRS
ML1292	8	19	12	0.978416667	DVQVPPSTYYTV
ML1292	66	76	11	0.897454545	LRSRQGDTADV
ML1344	36	57	22	1.516545455	WSRRAGTGPDTPAEGHGSRIDH
ML1384	97	112	16	1.288875	DGPDNPFGIGVGPGD
ML1384	58	69	12	1.005916667	ENRGAQAQGWKQ
ML1384	79	90	12	1.2435	SAGSGSSNIKPP
ML1445	11	20	10	1.1919	PQANKDGRRA
ML1575	2	12	11	0.905545455	MPWGSPGRWWS
ML1601	102	114	13	0.753769231	DDDLVTSVPPCGR
ML1604	17	26	10	1.2011	SDAPADEVVP
ML1605	39	54	16	0.8398125	GRFGPGIANPWTPIAD
ML1717	48	60	13	1.156615385	EVSPSSMNDNRGP
ML1788	3	18	16	1.3664375	DLSDSAAGPQSGPELG
ML1788	30	45	16	0.847625	QAQFNGQYQTGELGAH
ML1788	110	124	15	0.615133333	QRRAHRQVSSTEPIT
ML1915	45	55	11	1.298636364	SGHTDEQDDAL
ML1915	18	27	10	1.1588	GVATPQQTDQ
ML1915	83	92	10	0.5958	LYGAGPVKAV
ML1976	99	115	17	0.876764706	GRGEETLSANIPTSKSR
ML1976	50	63	14	0.818142857	ATTKPAIRPEISDA
ML1979	76	90	15	0.741466667	PIGSSSVSCVPEPNS
ML1979	48	57	10	0.8398	MSVPSADGAA
ML1989	77	102	26	1.162153846	KTTSNSPSDDLLHSNPQPEIYSTKST
ML2035	52	72	21	1.255190476	QEPNNGLVDPAAASSKPDWNP
ML2035	22	41	20	1.1337	VDGPRPSLPKSIDEGRWDRT
ML2044	36	45	10	0.8911	EQNTQAPAVR
ML2172	100	114	15	1.175066667	PSALGGPDQRTTRTA
ML2172	40	53	14	0.670285714	DRLEHNLPNSGWDC
ML2176	71	85	15	1.059	ATTPYTSEPVCSTQS
ML2249	79	90	12	0.823416667	SHAAAHAPQADK
ML2252	62	71	10	1.0663	DIDNDGSAVA
ML2284	67	86	20	0.98085	DSATAEVTSKEDNYSGVQVT
ML2284	105	119	15	0.988	GYPIYPPVPTIRKPS
ML2346	190	203	14	1.415785714	RKHSRYKPPNPARE
ML2346	157	168	12	1.065	SNVSSPGGAFDA
ML2347	159	174	16	1.17725	TWAGSDEHQHNAPSDL
ML2468	77	96	20	0.75735	ASSTDYRDHRAHQPVTLPQR
ML2468	103	121	19	1.225684211	HRRTEQSECTNSGGRPTTV
ML2562	13	26	14	0.992285714	AAEPCAPEGHRIEG

ML2562	71	84	14	1.259071429	LASGPAERADPCRP
ML2562	29	40	12	0.69525	HALPWPADRIQR
ML2567	62	88	27	1.136925926	SVTPASPPRKNHAVSSDFKTRSTNTEN
ML2567	136	157	22	1.105590909	LRQPDGSARHANHPFDQPSSRM
ML2567	105	126	22	1.524818182	PSNITEGTNASKSPQPAPTLTS
ML2567	37	53	17	1.188941176	QVRPGKPINNTNTTPAA
ML2651	36	48	13	0.746384615	EQAITPRTRSTIA
ML2666	9	30	22	1.464	LHPGPSSSTKNSTTSGNTAVTT
