# Supplementary figures and images for: Decoding the similarities and differences among mycobacterial species
Source: PLoS Negl Trop Dis. 2017 Aug 30;11(8):e0005883. doi: 10.1371/journal.pntd.0005883 (PMC5595346; doi:10.1371/journal.pntd.0005883)

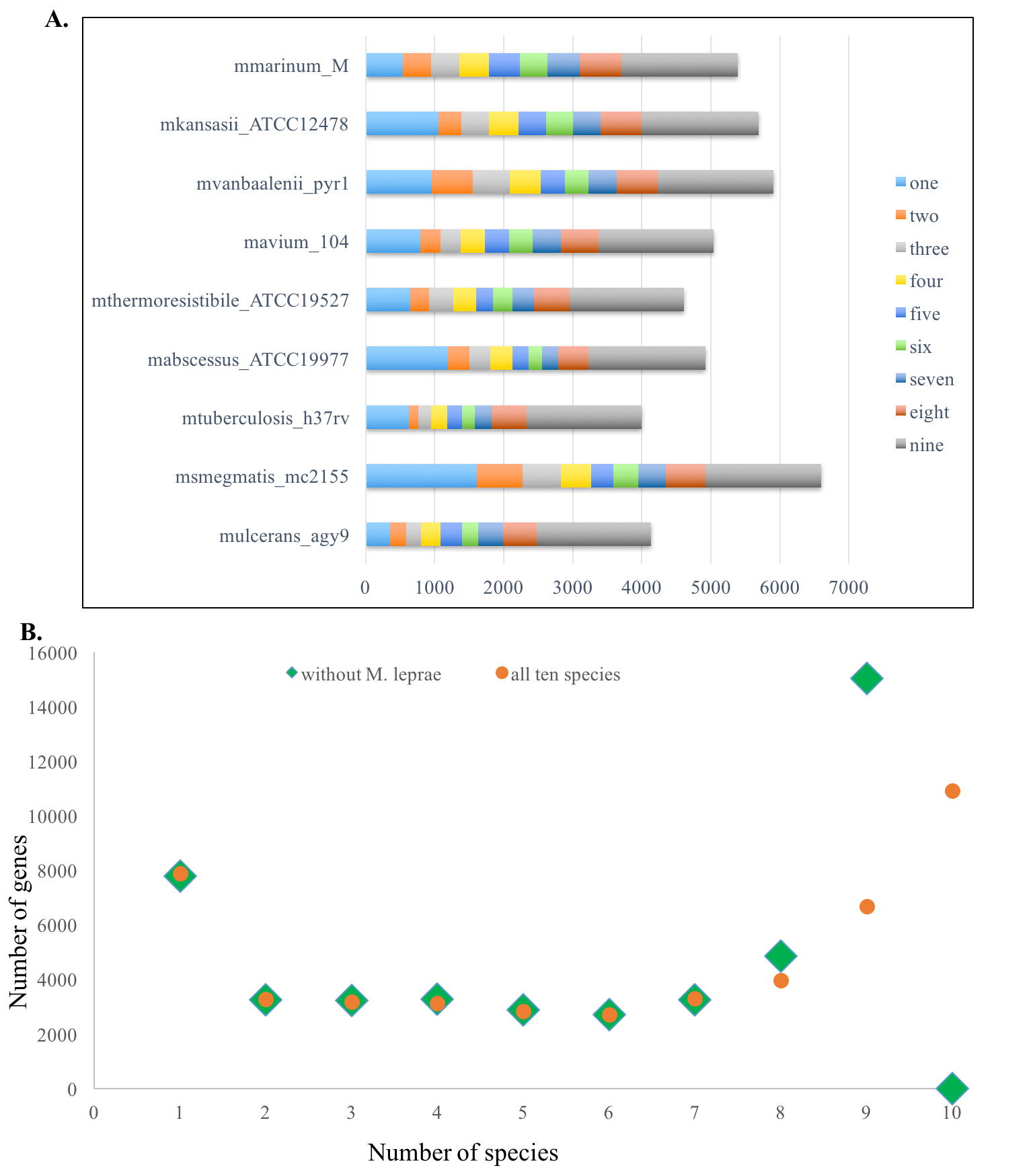

Supplement: S1 Fig — A. Species-wise representation of ortholog clusters. The bar proximal to the y-axis represents the proteins shared across all species, whereas the most distal bar represents the species-specific proteins. B. The number of genes present in the cluster that share a given number of species including and excluding M. leprae. (TIF) [file pntd.0005883.s008.tif]

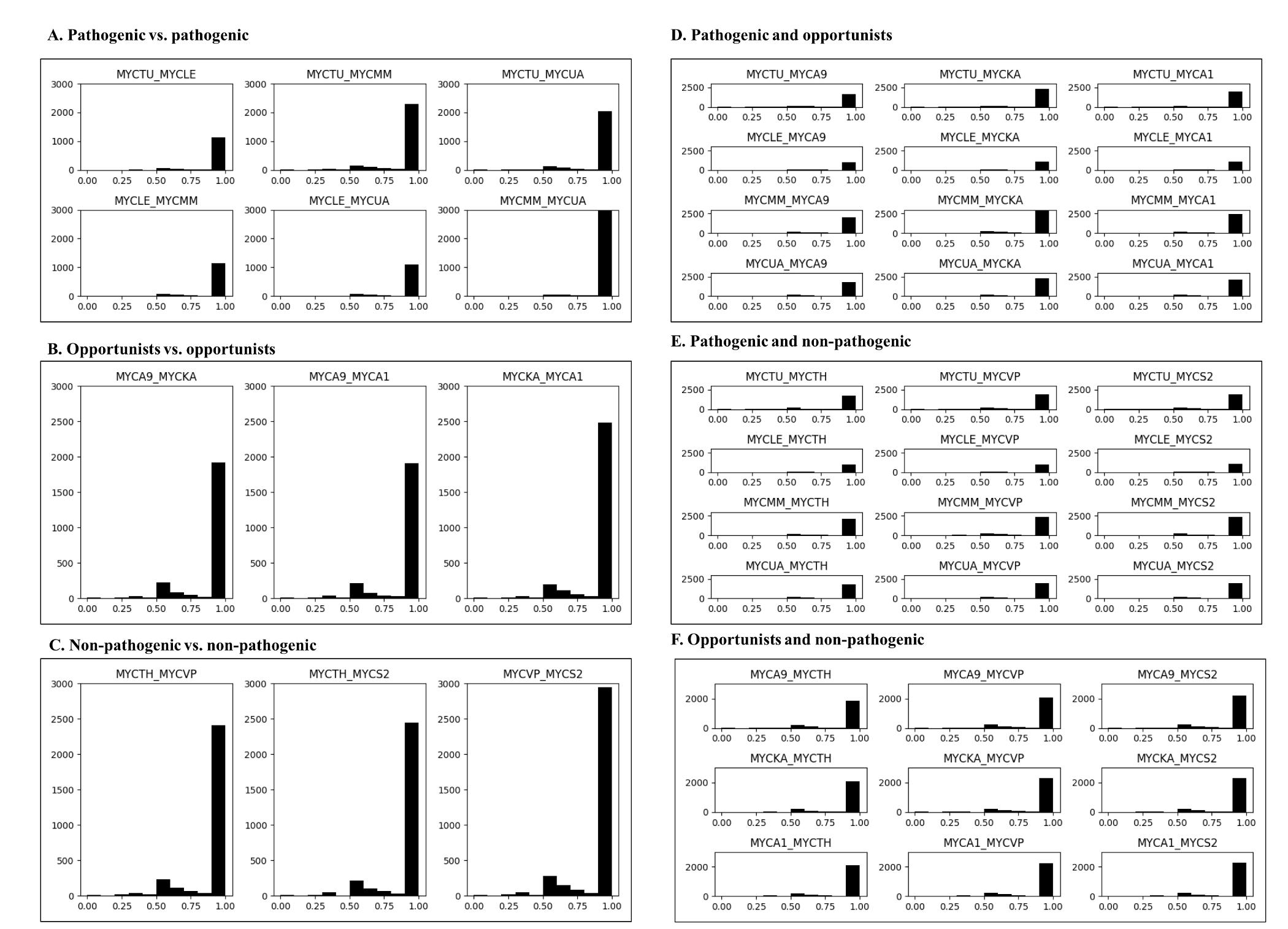

Supplement: S2 Fig — Domain composition scores for: A. Pathogenic vs. pathogenic species, all the scores mostly lie between 0.7–1.0, B. Opportunists vs. opportunists, C. Non-pathogenic vs. non-pathogenic. For B and C score below 0.5 are also seen. D. Pathogenic and opportunists- share high functional similarities, E. Pathogenic and non-pathogenic and F. Opportunists and non-pathogenic. MYCTU: M. tuberculosis, MYCS2: M. smegmatis, MYCUA: M. ulcerans, MYCA9: M. abscessus, MYCTH: M. thermoresistible, MYCA1: M. avium, MYCLE: M.leprae, MYCKA: M. kansasii, MYCVP: M. vanbaalenii, MTCMM: M. marinum. (TIF) [file pntd.0005883.s009.tif]

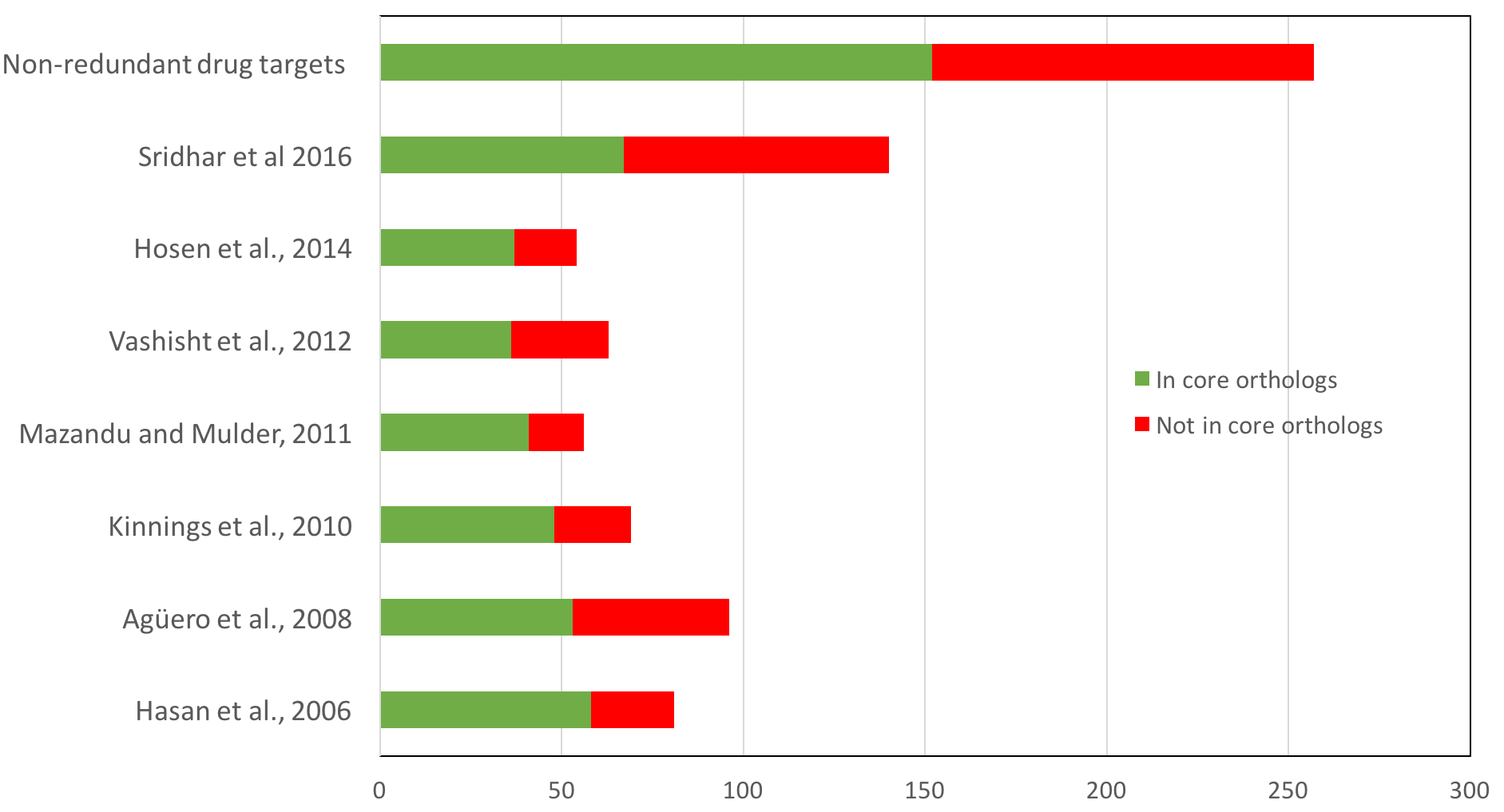

Supplement: S3 Fig — The representation of predicted targets for M. tuberculosis in other mycobacterial genomes. (TIF) [file pntd.0005883.s010.tif]

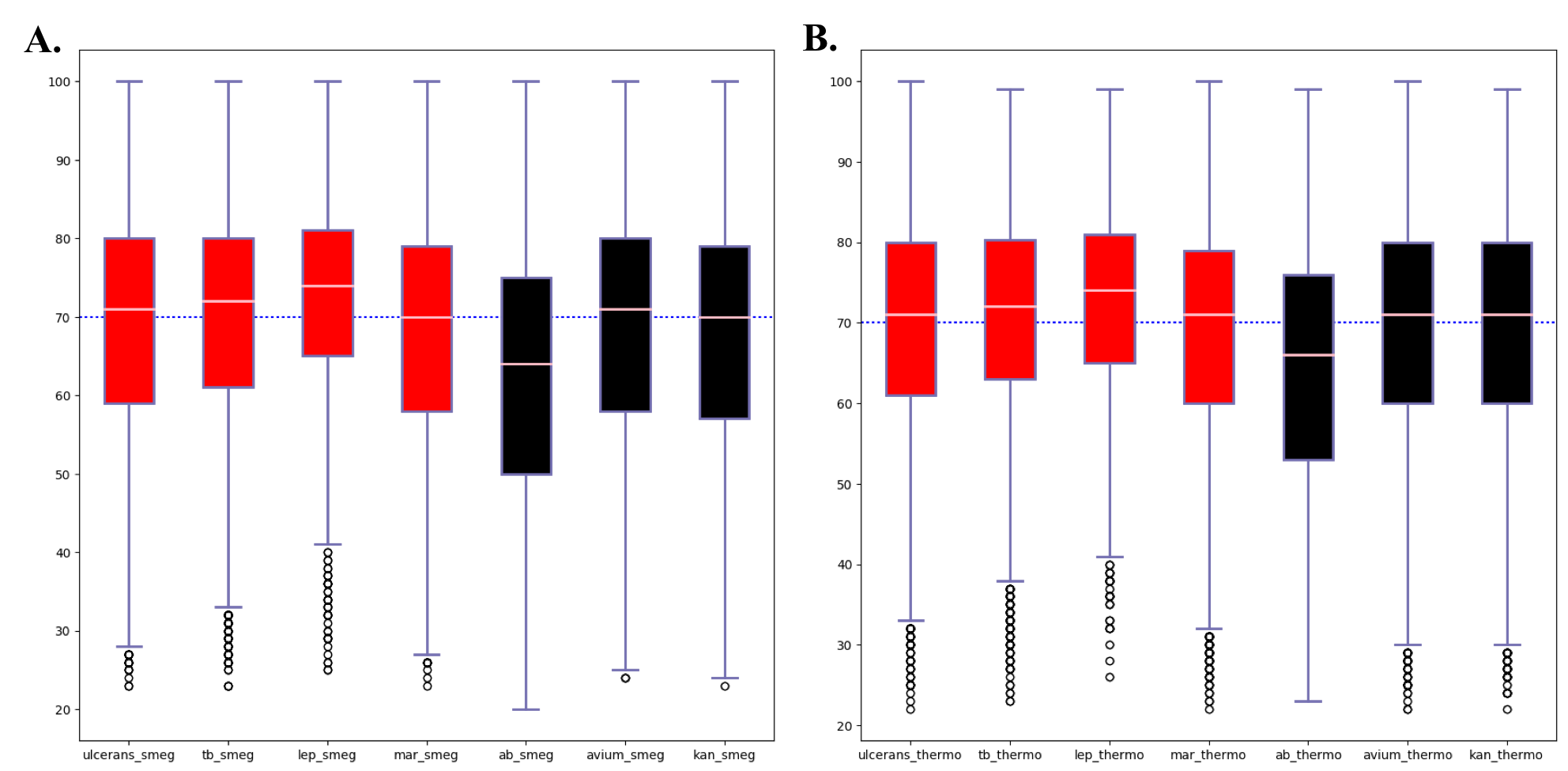

Supplement: S4 Fig — A. Box-plot showing the distribution of percent identities between the orthologs of pathogenic species (shown as red boxes) and orthologs of opportunist pathogenic species (shown as black boxes) with their orthologs in M. smegmatis genome. B. Box-plot showing the distribution of percent identities between the orthologs of pathogenic species (shown as red boxes) and orthologs of opportunist pathogenic species (shown as black boxes) with their orthologs in M. thermoresistibile genome. The abbreviations in the figure used are as follows: ulcerans_smeg: M. ulcerans vs. M. smegmatis, tb_smeg: M. tuberculosis vs. M. smegmatis, lep_smeg: M. leprae vs. M. smegmatis, mar_smeg: M. marinum vs. M. smegmatis, ab_smeg: M. abscessus vs. M. smegmatis, avium_smeg: M. avium vs. M. smegmatis, kan_smeg: M. kansasii vs. M. smegmatis. Similarly, the pairs with M. thermoresistibile (thermo). (TIF) [file pntd.0005883.s011.tif]

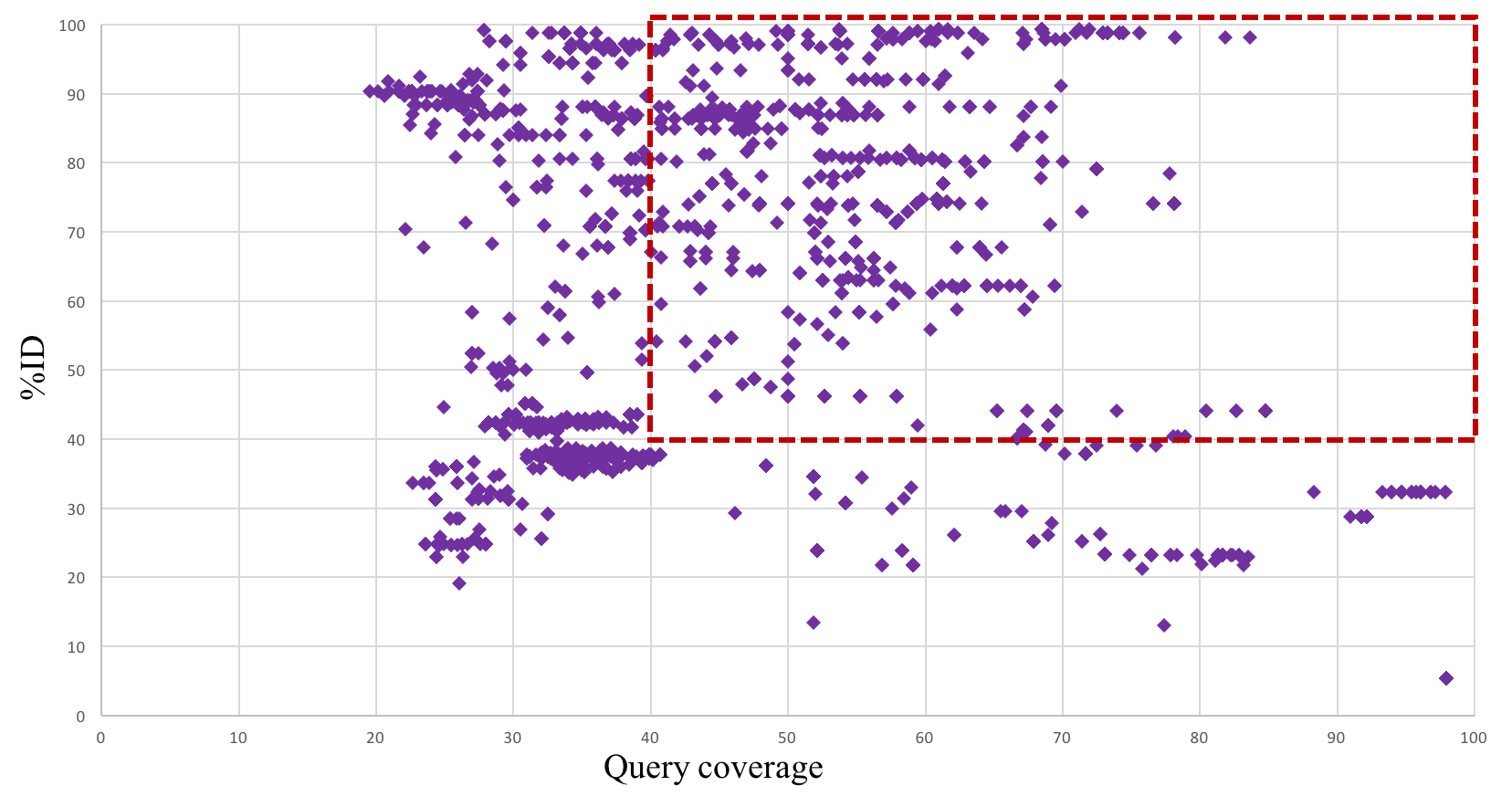

Supplement: S5 Fig — The proteins identified as specific in M. leprae genome (141 proteins) were searched against other mycobacterial species’ genomes. The alignment results are plotted as sequence identity vs. query coverage. The proteins which have a hit of at least 40% query coverage and 40% sequence identity were excluded from the M. leprae species specific set (marked in red rectangle, represents 55 proteins out of 141). The remaining proteins (86) were considered for further analysis. (TIF) [file pntd.0005883.s012.tif]
